# Tuning the Redox Chemistry of a Cr/SiO_2_ Phillips Catalyst for Controlling Activity, Induction Period and Polymer Properties

**DOI:** 10.1002/cphc.202000488

**Published:** 2020-07-06

**Authors:** Maarten K. Jongkind, Theo van Kessel, Marjolein E. Z. Velthoen, Nic. Friederichs, Bert M. Weckhuysen

**Affiliations:** ^1^ Inorganic Chemistry and Catalysis Debye Institute for Nanomaterial Science Utrecht University Universiteitsweg 99 3584 CG Utrecht The Netherlands; ^2^ SABIC Technology and Innovation Department Urmonderbaan 22 6167 RD Geleen The Netherlands

**Keywords:** Phillips catalyst, in-situ UV-VIS-NIR diffuse reflectance spectroscopy, olefin polymerization catalysis, chromium, polymerization

## Abstract

The Cr/SiO_2_ Phillips catalyst has taken a central role in ethylene polymerization ever since its discovery in 1953. This catalyst is unique compared to other ethylene polymerization catalysts, since it is active without the addition of a metal‐alkyl co‐catalyst. However, metal‐alkyls can be added for scavenging poisons, enhancing the catalyst activity, reducing the induction period and altering polymer characteristics. Despite extensive research into the working state of the catalyst, still no consensus has been reached. Here, we show that by varying the type of metal‐alkyl co‐catalyst and its amount, the Cr redox chemistry can be tailored, resulting in distinct catalyst activities, induction periods, and polymer characteristics. We have used in‐situ UV‐Vis‐NIR diffuse reflectance spectroscopy (DRS) for studying the Cr oxidation state during the reduction by tri‐ethyl borane (TEB) or tri‐ethyl aluminum (TEAl) and during subsequent ethylene polymerization. The results show that TEB primarily acts as a reductant and reduces Cr^6+^ with subsequent ethylene polymerization resulting in rapid polyethylene formation. TEAl generated two types of Cr^2+^ sites, inaccessible Cr^3+^ sites and active Cr^4+^ sites. Subsequent addition of ethylene also revealed an increased reducibility of residual Cr^6+^ sites and resulted in rapid polyethylene formation. Our results demonstrate the possibility of controlling the reduction chemistry by adding the proper amount and type of metal‐alkyl for obtaining desired catalyst activities and tailored polyethylene characteristics.

## Graphical abstract

**Role of Metal‐alkyls in Phillips catalysts**: Combining batch‐ethylene polymerization reactions with in‐situ UV‐Vis‐NIR diffuse reflectance spectroscopy experiments of an industrial Cr/SiO_2_ Phillips catalyst allowed for correlating the decreased induction period and increased catalyst activity to metal‐alkyl induced changes in the Cr reduction chemistry.

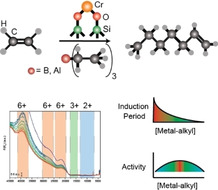

## 1 Introduction

Polyethylene (PE) is one of the most important polymers in today's society, with its annual production exceeding 80 million tons.^1^ The catalytic production of polyethylene heavily relies on three workhorses, namely Ziegler‐Natta type catalysts^2^, ^3^, (post‐) metallocene type catalysts^4^ and Phillips‐type catalysts.^1^, ^5^, ^6^, ^7^, ^8^ The Cr/SiO_2_ Phillips‐type catalyst is uniquely positioned with respect to the two other ethylene polymerization catalysts. Whereas Ziegler‐Natta and Group 4 transition metal based (post‐)metallocene catalyst types need to be activated by a metal‐alkyl co‐catalyst, this is not required for the Phillips catalyst. Here, ethylene can fulfil a dual role of activator and monomer source. On the other hand, metal‐alkyls can be added to scavenge poisons, to decrease the induction period, to enhance the ethylene polymerization rate, and to control the polymer product characteristics. Another remarkable difference is that, in contrast to Ziegler‐Natta and the (post‐)metallocene catalysts, the chain termination/transfer in Phillips‐type systems is only mildly sensitive to H_2_.^1^

Ever since its discovery by Hogan and Banks at Phillips Petroleum Company in 1953,^9^, ^10^ the Phillips catalyst is nowadays responsible for more than 30 % of all High‐Density Polyethylene (HDPE) produced world‐wide. Despite this catalyst's well‐established importance, there still is no consensus on its working state and on the related ethylene polymerization mechanism. Completely understanding the functioning of this catalyst is, however, of paramount importance for further fine‐tuning catalyst properties as well as for defining product characteristics.

Numerous research efforts have focused on understanding the active site oxidation state and molecular structure of this catalyst, which until date remains a matter of debate.^11^, ^12^, ^13^, ^14^, ^15^, ^16^, ^17^ Solving this problem remains challenging because of at least two reasons. First of all, only a very low Cr weight loading is viable for Phillips‐type catalysts, while secondly, this system is defined by a large variety among Cr sites of which only a small portion (maximum of ∼30 %) is proposed to be active in ethylene polymerization.^18^, ^19^, ^20^, ^21^, ^22^

This has resulted in the use of various characterization approaches often avoiding high pressures and temperatures. Examples include model systems^22^, ^23^, ^24^, ^25^, ^26^, ^27^, ^28^ or well‐defined catalyst systems in which Cr^6+^ is reduced by e. g. CO.^13^, ^14^, ^29^, ^30^, ^31^ With an array of spectroscopic techniques revealing that the oxidation state of the Cr active site lies between 2 and 3.^13^, ^14^, ^20^, ^32^, ^33^, ^34^, ^35^, ^36^, ^37^, ^38^, ^39^, ^40^, ^41^, ^42^, ^43^, ^44^, ^45^ However, assigning a definitive oxidation state number remains a matter of debate due to the various findings from different groups, although this could be in part explained due to different catalyst materials and reaction set‐ups.^6^, ^20^, ^34^, ^36^, ^37^, ^38^, ^40^, ^41^, ^42^, ^46^, ^47^, ^48^, ^49^

Fortunately, with the continuous development of advanced spectroscopic and microscopic techniques, the low Cr weight‐loading and relatively small portion of active sites are becoming less of a problem, exemplified by elucidation of an ethylene reduced active site.^18^ Even though our understanding of CO and ethylene reduced Cr/SiO_2_ catalysts is increasing, research towards the effects of metal‐alkyl co‐catalysts on the active site structure and oxidation state has only started to gain track more recently. With one of the latest examples demonstrating the effect of TEAl on the Cr/SiO_2_ catalyst and revealing the presence of bis‐grafted bis‐alkyl Cr^4+^ species as the ethylene polymerizing sites, coordinatively saturated Cr^3+^ sites as inactive sites as well as two types of mono‐grafted mono‐alkyl Cr^2+^ sites.^50^

These recent findings were preceded by work from our group: investigating the effect of TEAl on an industrial shell‐titanated Cr/Ti/SiO_2_ Phillips‐type catalyst, also demonstrating the enhanced degree of *α*‐oligomer generation and incorporation^51^ as well as a significantly enhanced polymerization activity.^52^ In addition, Scanning Transmission X‐ray Microscopy (STXM) was used to reveal two different types of active sites, namely an ethylene polymerizing active site, which polymerized ethylene following the Cossee‐Arlman mechanism and ethylene oligomerizing sites, which produced ethylene oligomers via the metallacycle mechanism. The properties of the active sites, in this research, being determined by either the titanium‐rich environment in the shell or the titanium‐lean inner environment: the former producing a more linear PE and the latter a more branched PE.^53^ These examples from our group as well as those from Groppo et al. demonstrate that a lot of insights with respect to the effect of co‐catalysts on the Cr/SiO_2_ catalyst are to be gained and it is exactly this field of research that is paramount for optimizing existing ethylene polymerization processes and for finding new routes towards (new) polyethylene materials.

Continuing on this research effort into investigating the influence of metal‐alkyl co‐catalysts on the surface chemistry, and the working state of the Cr/SiO_2_ Phillips ethylene polymerization catalyst, we here study the influence of tri‐ethyl aluminum (TEAl) and tri‐ethyl borane (TEB) on the oxidation state during reduction and during polymerization, on the induction period, on the catalyst activity and on the polymer characteristics. In a first series of experiments we opted to investigate how TEB and TEAl affect bulk properties, such as the catalyst activity, induction period, and, polymer characteristics, on an industrial 1 wt% Cr/SiO_2_ Phillips catalyst. As a consequence of the observed unique catalytic performances, we have investigated how the different metal‐alkyl co‐catalysts affect the reduction chemistry of the Cr/SiO_2_ Phillips catalyst with in‐situ UV‐Vis‐NIR DRS. The combination of experimental techniques revealed that TEB and TEAl uniquely interact with the Cr/SiO_2_ Phillips catalyst, resulting in specific reduction pathways and bulk performances.

## 2 Results and Discussion

### 2.1 Metal‐Alkyl Influence on Induction Period and Catalyst Activity

**Figure 1:**
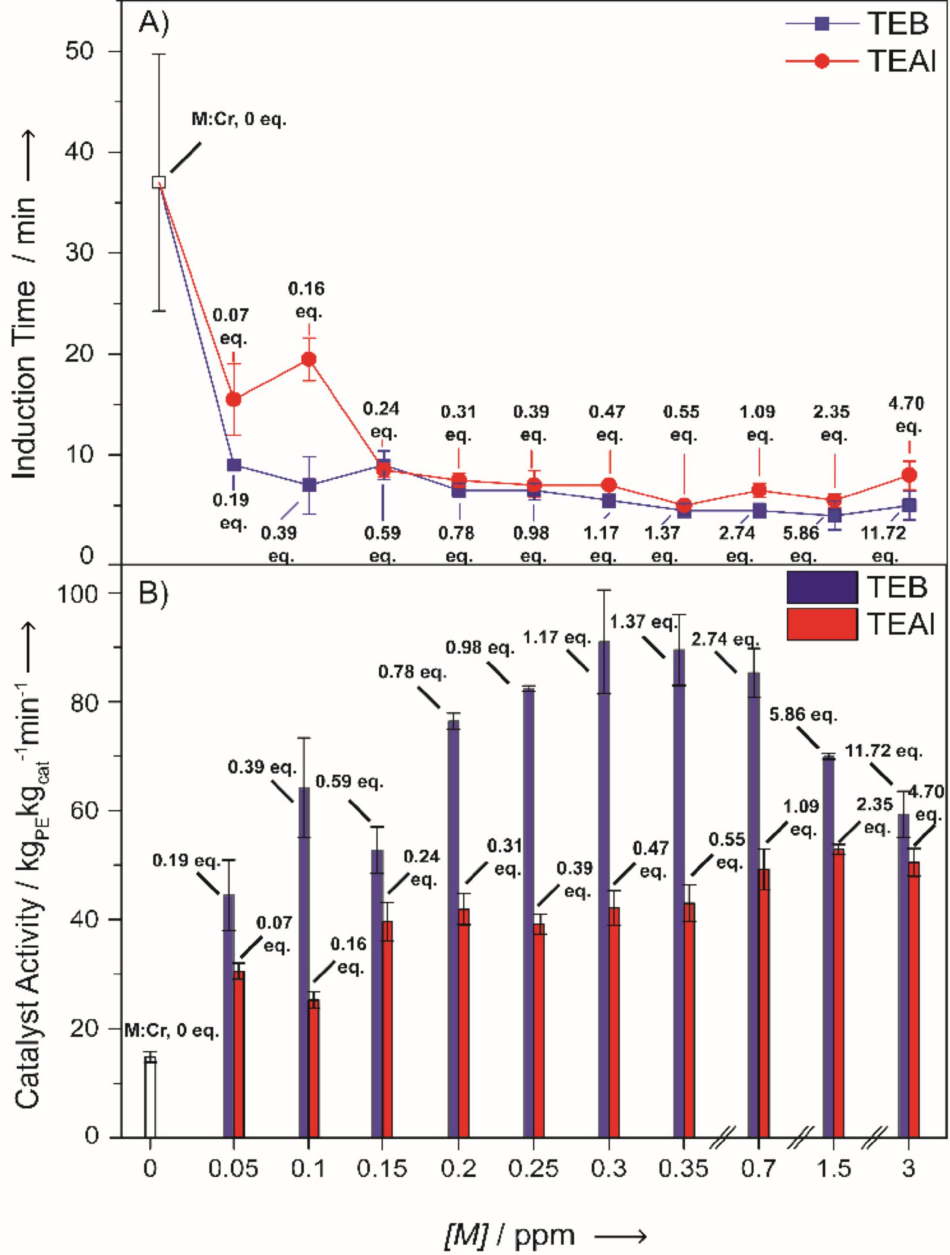
The effect of TEB (blue) or TEAl (Red) on A) the induction periods and B) the catalytic activities during the batch ethylene polymerization reactions. All the reactions were performed in a 5 L semi‐batch ethylene polymerization reactor, at 34 bar and 99 °C. The M : Cr mole ratios are given in both figures, with lower values for TEAl than for TEB due to the larger MW for TEAl.

Figure 1 illustrates the influence of TEAl and TEB on the induction period and the catalyst activity during the performed semi‐batch ethylene polymerization reactions, with ppm (wt/wt) reflecting the added amount of the co‐catalyst to the diluent. Prior to ethylene polymerization, the Cr^6+^/SiO_2_ Phillips catalyst has to be reduced to its active state, the duration of this process is defined as the induction period. Practically, the length of this period was determined as the time between the first contact of the catalyst material with ethylene and the moment at which ethylene had to be actively added for maintaining the reactor pressure. One of the benefits of metal‐alkyl co‐catalysts is that they already reduce a number of the Cr^6+^ surface sites and as a consequence partly remove the need for reduction by ethylene, resulting in a strongly decreased induction period. In addition, poisonous oxygen‐containing compounds, originating either from impurities in the gas feed or reduction by‐products, are also removed by these co‐catalysts, preventing the re‐oxidation and/or poisoning of Cr active sites. These two features combined ensure a more reliable reduction process.

The importance of the co‐catalyst in reducing the induction period is demonstrated in Figure 1A, in the presence of 0.05 ppm of TEB (0.19 B : Cr mole ratio) it was decreased to 10 min and with 0.05 ppm of TEAl (0.07 Al : Cr mole ratio) it was decreased to 15 min. The induction period was decreased to a larger extent, until 0.15 ppm with TEB (0.59 B : Cr mole ratio) as compared to TEAl (0.24 Al : Cr mole ratio). However, beyond these co‐catalyst amounts the induction periods converged to a minimum of 8 min. Apparently, a smaller mole ratio is required to attain the minimum induction period of 8 min for TEAl as compared to TEB. The observed increase in induction period at 0.1 ppm TEAl (0.16 Al : Cr mole ratio) is attributed to an artefact in measuring the induction period.

Figure 1B illustrates how the incremental amounts of TEB and TEAl affect the catalytic activities. Addition of either co‐catalyst is beneficial for the catalyst activity. However, TEB raises the activity to a larger extent than TEAl does, which is testified by the three‐fold activity increase in the presence of TEB whereas TEAl only results in an activity increase by a factor 2. The catalyst activity passes through a maximum of 90 kg_PE_ kg_cat_^−1^ min^−1^ in the presence of 0.30 ppm TEB (1.17 B : Cr mole ratio), whereas the catalyst activity in the presence of 0.30 ppm TEAl (0.47 Al : Cr mole ratio) is 40 kg_PE_ kg_cat_^−1^ min^−1^. Interestingly, nearly equimolecular amount of TEB or TEAl (0.30 ppm, 1.17 B : Cr mole ratio; 1.09 Al : Cr mole ratio) did not result in converged catalyst activities, instead the catalyst activity with TEAl was still approximately half the catalyst activity with TEB. By further increasing the amount of TEB the activity starts to decrease again, this is most evident with amounts of TEB above 1.50 ppm (5.86 B : Cr mole ratio). McDaniel et al. already reported that TEB amounts above 5.0 ppm negatively affect the catalyst activity and that this is most likely caused by over‐reduction of the catalyst material.^1^, ^54^, ^55^ On the other hand, the presence of TEAl does not seem to be disadvantageous for the catalyst activity for any of the used amounts, as is demonstrated by the relatively constant catalyst activity. However, a likely explanation is that we have not crossed the threshold for which catalyst performances start to decrease.

Table 1 gives an overview of the effect TEB and TEAl on selected polymer characteristics and structural parameters of the PE products from the semi‐batch ethylene polymerization reactions. Detailed information for all semi‐batch ethylene polymerization experiments is provided in the Supporting Information (Tables S2, S3 and S4). The Melt Flow Index (MFI) represents the polymer molecular weight and gives an indication of the (weight averaged) molecular weight of the polyethylene, as further evidenced by the correlation between the MFI5 and the Mw. The Melt Flow Index Ratio (MFIR), defined as MFI_21.6_/MFI_5_, gives an indication of the rheological broadness of the polymer. Both co‐catalyst materials lower the polymer MW and increase the rheological broadness of the produced PEs. However, TEB appears to do so more significantly, since the MFIR increased by over 50 % already by the addition of only 0.30 ppm TEB (1.17 B : Cr Mole Ratio) whereas a 25 % increase of the MFIR is observed in the presence of the same TEAl concentration (0.47 Al : Cr Mole Ratio). In terms of Long Chain Branching (LCB) we see that for similar Mw/Mn values the MFIR (MFI21.6/MFI5) is higher for TEB than for TEAl, possibly indicating an enhanced degree of LCB for the polymers produced with TEB, where LCB is defined as a side‐chain of more than 150 C atoms. For example, the MFIR at a Mw/Mn of ∼29.3 is 28.8 for TEB and 26.0 for TEAl, further exemplified in Figure S1.

**Table 1:** An overview of the polymer product ratios from the products obtained from the 5 L batch ethylene polymerization reactions: Melt Flow Index (MFI) 5 and MFI 21.6, the Melt Flow Index Ratio (MFIR), Polymer Density (PD), Powder Bulk Density (PBD), D50 Particle Size (PSE) and Particle Span after ethylene polymerization. Analysis of the produced PE materials with Gel Permeation Chromatography – Size Exclusion Chromatography – Differential Viscometry – Infrared (GPC‐SEC‐DV‐IR) provided the Mn, Mw, Mz structural parameters.

Exp.	M : Cr Mole Ratio	MFI 5 [dg min^−1^]		MFI 21.6 [dg min^−1^]	Melt Flow Index Ratio (MFIR)	Polymer Density [kg m^−3^]	Powder Bulk Density [kg m^−3^]	PSE D50 [μm]	Span (D90‐D10)/D50	Mn [kDa]	Mw [kDa]	Mz [kDa]	Mw/Mn
Cr/SiO_2_	0	0.13		2.7	20.8	956.1	442	636	0.87	19	360	2600	18.6
**TEB**													
0.30 ppm	1.17	0.26		8.5	32.7	960.0	268	464	1.10	11	330	3000	30.3
1.50 ppm	5.86	0.43		12.4	28.8	958.8	254	504	1.12	11	220	1700	20.8
3.00 ppm	11.72	0.32		10.9	34.1	958.9	271	582	0.96	9	270	2400	29.1
**TEAl**													
0.30 ppm	0.47	0.13		3.3	25.4	954.6	373	490	1.28	16	370	2900	23.6
1.50 ppm	2.35	0.25		6.5	26.0	953.4	380	472	1.24	12	340	2900	29.3
3.00 ppm	4.71	0.32		7.7	24.1	953.5	362	401	1.31	12	320	2900	28.0

The obtained polymer densities give an indication of polymer crystallinity, which is predominantly governed by Short Chain Branching (SCB) and molecular weight.^56^ Interestingly, here we see that the presence of TEB resulted in a polymer with increased density in combination with an increased MFIR. Polymerization reactions in the presence of TEAl produced PEs with decreased density, while the MFIR still increased. This decreased polymer density infers the presence of SCB as a result of C_2_H_4_ oligomerization and increased *α*‐olefin incorporation.

The Powder Bulk Density (PBD) is affected by the powder morphology, like for instance particle shape and particle size distribution. Table 1 indicates that in the absence of a metal‐alkyl co‐catalyst a PBD of 400 kg/m^3^ is obtained, and that this value is highly dependent on the type of co‐catalyst that is added and less so on the added amount. Addition of TEB resulted in a decreased PBD, with a minimum of 257 kg/m^3^ in the presence of 1.50 ppm (5.86 B : Cr mole ratio). In contrast, with 3.00 ppm TEAl (4.71 Al : Cr mole ratio) a minimum PBD of 360 kg/m^3^ was observed.

An increase of the particle size distribution broadness after ethylene polymerization, as compared to polymerization without co‐catalyst, is an indication of increased particle fragmentation. Careful inspection of the PSE D50 and Span in Table 1 reveals that addition of either co‐catalyst increases the span and PSE D50. However, the broadness is increased to a larger extent in the presence of TEAl than in the presence of TEB. In case the polymer powder particles obey the replication phenomenon, the average particle size (expressed as D50) is governed by the catalyst yield. In these experimental series, the catalyst yield was nearly constant, which allows a discussion on the effect of the co‐catalyst on the particle size of the polymer. Quite interestingly, both co‐catalysts cause an initial decrease in D50 and increase of the particle span compared to the polymer without TEB or TEAL. 0.30 ppm of TEB (1.17 B : Cr mole ratio) produces particles with a D50 of 464 μm and a particle span of 1.10, which subsequently changes to a value of 582 *μ*m and a particle span of 0.96 with 3.00 ppm of TEB (11.72 B : Cr mole ratio). In the case of TEAL however, the D50 decreases to a value of 401 *μ*m and a particle span of 1.31 with 3.00 ppm of TEAl (4.71 Al : Cr mole ratio). This infers that incremental amounts of TEAl enhance particle fragmentation, whereas incremental amounts of TEB suppress particle fragmentation.

**Figure 2:**
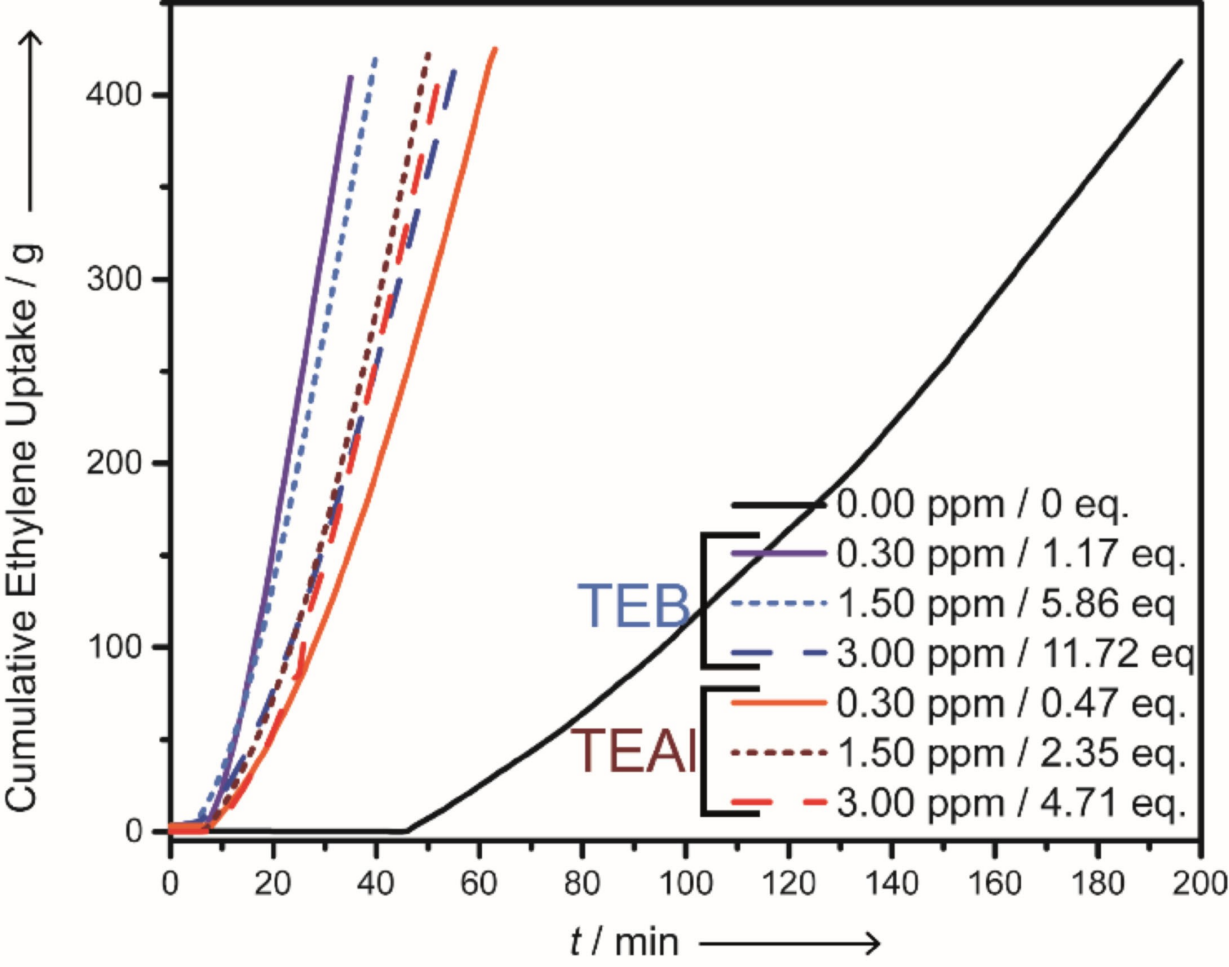
An overview of the kinetic profiles obtained by measuring the cumulative ethylene uptake during the semi‐batch ethylene polymerization reactions at 99 °C and 34 bar. The blue lines indicate the reactions performed with TEB and the red lines respectively the reactions with TEAl.

Figure 2 demonstrates a significantly enhanced kinetic profile in the presence of 0.30 ppm TEB (1.17 B : Cr mole ratio), whereas the polymerization rate decreases upon further increasing its amount. TEAl, on the other hand, results in an enhanced kinetic profile starting from 0.30 ppm (0.47 Al : Cr mole ratio) to 1.50 ppm (2.35 Al : Cr mole ratio) and 3.00 ppm (4.71 Al : Cr mole ratio), whereas the last two are similar. These developments are in line with the similar trends observed for the bulk activities. Furthermore, almost equal kinetic profiles are observed for 3.00 ppm of either co‐catalyst material (11.72 B : Cr mole ratio; 4.71 Al : Cr mole ratio). By inspecting the kinetic profiles and the fragmentation patterns we observe that an increase in the former does not translate necessarily into an increase in the latter. Instead, fragmentation patterns are predominantly governed by the type of co‐catalyst rather than the measured kinetic profile.

### 2.2 Controlling the Cr Reduction Chemistry by Rational Co‐Catalyst Selection

In a second series of experiments the influence of the type, and amount of metal‐alkyl co‐catalyst on the Cr oxidation reduction chemistry was investigated. For this purpose, a series of UV‐Vis‐NIR DRS experiments were designed for studying oxidation state changes during reduction as well as subsequent polymerization with separation of these two stages in mind.

The in‐situ set‐up allowed for the reduction of the catalyst material by gaseous ethylene, or injection of a metal‐alkyl co‐catalyst solution into the N_2_ gas‐stream *via* the septum. A detailed scheme of the experimental set‐up is provided in Scheme S1. In order to investigate the effect of equimolar amounts of the different co‐catalysts on the reduction chemistry, of which the results on the different reductants will be discussed as follows:

- 1.50 mole ratio of TEB (B : Cr)
- 1.50 mole ratio of TEAl (Al : Cr)
- 10.0 mole ratio of either co‐catalyst (M : Cr)

Additionally, the Supporting Information provides a series of reference material UV‐Vis‐NIR DRS spectra, in Figures S2 and S3, which were also subjected to spectral deconvolution. The Supporting Information also contains the spectral deconvolution process on the materials reduced with TEB or TEAL (Figures S6, S8, S10, S12, S14 and S16), this will be discussed at a later stage.

#### 2.2.1 Ethylene Polymerization after Pre‐Treatment with 1.50 Molecular Equivalents of Tri‐Ethyl Borane

**Figure 3:**
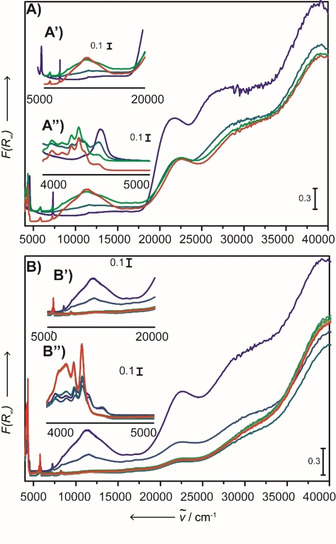
UV‐Vis‐NIR DRS spectroscopic developments for the UV‐Vis‐NIR DRS experiment with a B : Cr mole ratio of 1.50. A) The UV‐Vis‐NIR DRS spectroscopic developments during pre‐treatment of the catalyst with 1.50 molecular equivalents of TEB under an N_2_ stream of 10 mL min^−1^ at room temperature and ambient pressure, aiming for a B : Cr mole ratio of 1.50. The spectra, from blue to red, are recorded with 5 min intervals. With the blue spectrum representing the pristine catalyst. B) Developments after the N_2_ stream was switched to an ethylene stream of 10 mL/min, where the spectra were recorded at 20 °C intervals and the cell was heated to 150 °C with 5 °C min^−1^. The spectra, from blue to red, are recorded with 5 min intervals.

Firstly, we investigated the role of TEB, of which the results are shown in Figure 3. Figure 3A demonstrates the reduction at room temperature, where 1.50 mol. eq. TEB was added through the septum. Figure 3B illustrates ethylene polymerization while heating to 150 °C.

Directly after injection, a decrease in intensity of the Cr^6+^ Charge Transfer (CT) bands at ∼37500, ∼27500 and ∼22500 cm^−1^ is observed while a d‐d transition band at 11000 cm^−1^ developed. While the intensity of the O→Cr^6+^ CT bands decrease over time, their characteristic fingerprint remains visible throughout the entire reduction stage, inferring that not every Cr^6+^ site is reduced by TEB. The persistence of these bands makes it difficult to assign definitive oxidation state numbers. The color change from orange to pale yellow/green, as shown in Figure S16, is a direct consequence of the present CT bands with the newly emerging band at 11000 cm^−1^.^13^, ^14^, ^15^, ^29^

The absence of an absorption band at 16000 cm^−1^ might lead to the conclusion that no Cr^3+^_Oh_ species is formed, this is however rapidly dismissed by deconvoluted bands at 15500, 21500 and 33900 cm^−1^ shown in Figure S6. Furthermore, the d‐d transition band at 11000 cm^−1^ infers the presence of Cr^2+^ centers, with deconvoluted bands contributing respectively at 9400 and 11900 cm^−1^. The maximum of this Cr^2+^ d‐d transition band is, however, blue shifted in comparison to the d‐d transfer band generated by CO reduction (Figure S3). This at least excludes the presence of exclusively naked Cr^2+^ ions and indicates that reduction by‐products remain in the proximity of the Cr active sites, hereby affecting the d‐d transition and CT bands.^16^, ^57^, ^58^, ^59^, ^60^, ^61^, ^62^, ^63^ In order to ensure a proper fit, an additional band was required at 24000 cm^−1^, which is expected to be a CT band. This 24000 cm^−1^ CT band, which is absent in the CO reduced material, can be explained by reduction by‐products remaining in the coordination sphere of the Cr active site: affecting the location the d‐d transition bands and the CT bands.

Subsequently, the gas stream was switched from N_2_ to ethylene, followed by heating to 150 °C, with spectra being recorded at 20 °C intervals (5 min), as shown in Figure 3B. Changing the gas mixture resulted in an immediate decrease of the CT bands as well as the 11000 cm^−1^ band, inferring that the latter band is participating in ethylene polymerization. Ethylene polymerization, in turn, was confirmed by the emerging PE fingerprint, the formation of a white rubbery material and the loss of Cr related spectroscopic information due to lack of diffuse scattered UV‐Vis light caused by the PE layer around the Cr surface sites: hereby shielding them from detection.

#### 2.2.2 Ethylene Polymerization after Pre‐Treatment with 1.50 Molecular Equivalents of Tri‐Ethyl Aluminum

**Figure 4:**
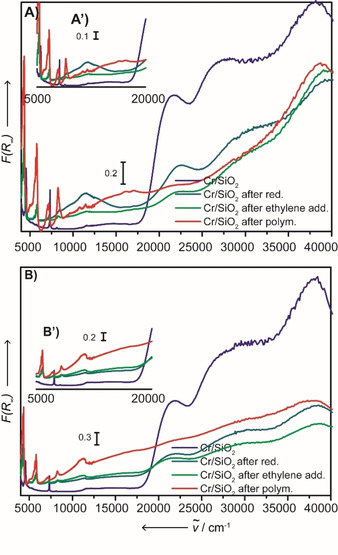
UV‐Vis‐NIR DRS spectroscopic developments for the UV‐Vis‐NIR DRS the experiment with an Al : Cr mole ratio of 1.50 A) The UV‐Vis‐NIR DRS spectroscopic developments during pre‐treatment of the catalyst with 1.50 molecular equivalents of TEAl under an N_2_ stream of 10 mL min^−1^ at room temperature and ambient pressure, aiming for an Al : Cr mole ratio of 1.5. The spectra, from blue to red, are recorded with 5 min intervals. With the blue spectrum representing the pristine catalyst. B) Developments after the N_2_ stream was switched to an ethylene stream of 10 mL min^−1^, where the spectra were recorded at 20 °C intervals and the cell was heated to 150 °C with a ramp of 5 °C min^−1^. The spectra, form blue to red, are recorded with 5 min intervals.

The results of the experiments with 1.50 molecular equivalents of TEAl are shown in Figure 4. The most recent work by Groppo et al.^50^ reported on the use of TEAL as a co‐catalyst. Their work revealed the existence of bis‐grafted bis‐alkyl Cr^4+^, inaccessible Cr^3+^ and two mono‐grafted mono‐alkyl Cr^2+^ surface sites. Moreover, it is worth stating that our findings on the UV‐Vis‐NIR DRS experiments match theirs, with (some) additional insights being provided due to the ability to perform reduction and polymerization in situ.

Directly after injection, as seen in Figure 4A, the Cr^6+^ CT bands decrease in intensity and a slight increase in intensity is observed from 20000 cm^−1^ to lower wavenumbers. Figure 4A also demonstrates a slight shift of the 22500 cm^−1^ band to lower wavenumbers, confirmed to be caused by the formation of the bis‐grafted bis‐alkyl Cr^4+^ species, with deconvolution (Figure S12) showing a contributing band at 19800 cm^−1^. Furthermore, the increase in the 20000–8000 cm‐1 region is attributed to the formation of two mono‐grafted mono‐alkyl Cr^2+^ surface sites (band contributing at 11000 cm^−1^) and one inaccessible Cr^3+^ site (band contributing at 15200 cm^−1^).^50^

One additional effect of TEAl is only revealed 15 min after exposure to ethylene, when bands at 9000 and 15500 cm^−1^ rapidly emerge and then disappear, all while CT bands decrease in intensity. This event indicates the reduction of Cr^6+^ species by ethylene, likely into Cr^2+^/Cr^3+^ similar to those of the ethylene reduced system, considering the matching locations of their bands upon deconvolution. These results demonstrate that TEAl not only acts as a reductant, but also as a reducibility enhancer. Subsequent polymerization was evidenced by loss of all Cr related spectroscopic information, the emerging PE fingerprint in the NIR region as well as the white reaction product.

#### 2.2.3 Ethylene Polymerization after Pre‐Treatment with 10 Molecular Equivalents of Co‐Catalyst

**Figure 5:**
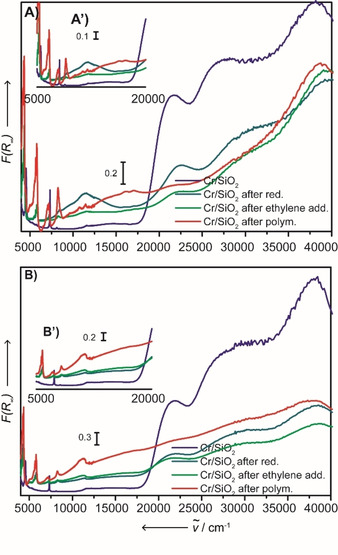
Spectra of (from blue to red) (Blue) the Cr/SiO_2_ catalyst before reduction with 10 molecular equivalents of co‐catalyst, (Dark Green) 15 min after reduction, (Light Green) 5 min into ethylene polymerization and (Red) after reaching 150 °C, after 25 min of ethylene exposure. A) displays the key UV‐Vis‐NIR DRS spectra with TEB as co‐catalyst, aiming for a B : Cr mole ratio of 10 : 1 and B) displays the key UV‐Vis‐NIR DRS spectra with TEAl, aiming for a Al : Cr mole ratio of 10.

Last, the bulk polymerization experiments revealed different catalyst performances if excesses of TEB or TEAl were used, therefore the experiments were repeated with 10 molecular equivalents co‐catalyst, for which key‐spectra are reported in Figure 5.

Interestingly, the addition of 10 molecular equivalents of TEB, shown in Figure 5A, appeared to alter the reduction pathway only to a small extent, testified by same trend where the CT bands decrease in intensity while a broad band at 11000 cm^−1^ emerged, with deconvolution (Figure S10) revealing bands contributing at 9400 and 11900 cm^−1^, all while retaining the CT fingerprint. Again, albeit Cr^3+^_Oh_ seems absent, its presence is confirmed by a band contributing at 16000 cm^−1^. A switch of the gas‐feed to ethylene demonstrated the polymerization properties by the loss of all Cr related spectroscopic information, the emerging PE fingerprint and the formation of a white rubbery material.

On the other hand, an excess amount of TEAl did affect the UV‐Vis‐NIR DRS spectra, as shown in Figure 5B. The excess co‐catalyst resulted in a more predominant increase in the 20000–8000 cm^−1^ region, with bands contributing at 15200 and 11100 cm^−1^ upon deconvolution (Figure S16). Furthermore, the 22500 cm^−1^ band again shifted, caused by the formation of bis‐grafted bis‐alkyl Cr^4+^ surface sites, deconvolution demonstrating a band contributing at 19800 cm^−1^ (Figure S16). These observations indicate the formation of additional Cr^4+^ sites from the reduction of Cr^6+^ sites, while simultaneously producing a larger number of Cr^2+^ and Cr^3+^ sites from the overreduction of these Cr^4+^ sites.^50^ Lastly, changing the gas stream to ethylene resulted in a small increase in intensity in the d‐d transition region, combined with a decrease in intensity of the CT region. This infers that the larger amount of TEAl suppressesthe subsequent ethylene reduction, likely due to less Cr^6+^ sites being available for this. Eventually, all Cr spectroscopic features disappeared, until the UV‐Vis‐NIR DRS spectrum was completely dominated by the PE fingerprint.

#### 2.2.4 How the Metal‐Alkyl Co‐Catalysts Affect the Degree of Reduction

Deconvolution of the UV‐Vis‐NIR DR spectra was performed according to the same, proven, method reported by Weckhuysen et al.^7^, ^13^, ^64^ while refraining from commenting on the active site structure, since this would require the use of additional techniques such as NEXAFS, probe molecule FTIR experiments and electron Paramagnetic Resonance (EPR) experiments. The established literature does allow for an in‐depth and semi‐quantitative discussion on the observed reduction chemistry.

The consistency of band centers and HWHMs during deconvolution validated our approach, of which the deconvolution parameters can be found in Supporting Information Tables S5–S8. Absolute quantification of the reduced Cr species in the UV‐Vis‐NIR DRS experiments is impossible for two reasons: (1) only for weight‐loadings between 0 and 0.2 wt% a linear relationship between Cr^6+^ band intensity and Cr^6+^ concentration exists and (2) knowledge of the exact absorption coefficient is required.^13^ However, Equation (1**)** does show that semi‐quantification is possible without knowing the exact extinction coefficients, since *“ϵ*_*reduced‐species*_ */ϵ_chromate_”* is reduced to a constant: K. Therefore, the ratios between band intensities from reduced Cr species in the d‐d transition region and from the 31000 cm^−1^ Cr^6+^ band remain a semi‐quantitative measure of the reduction.

**Figure 6:**
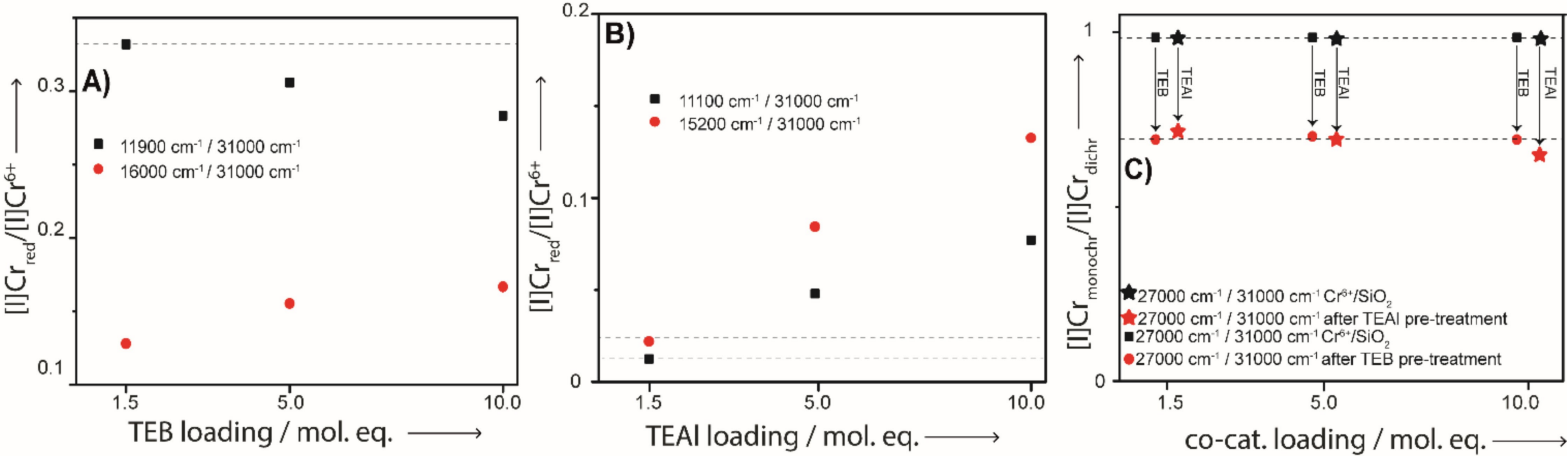
A) The band intensity ratios from the deconvoluted UV‐Vis‐NIR DRS spectra. The 11900 cm^−1^/31000 cm^−1^ band ratio and 16000 cm^−1^/31000 cm^−1^ band ratios are shown for the experiments with TEB. B) The band intensity ratios from the deconvoluted UV‐Vis‐NIR DRS spectra. The 11100 cm^−1^/31000 cm^−1^ band and 15200 cm^−1^/31000 cm^−1^ band ratios are shown for the experiments with TEAl. The 19800 cm^−1^/31000 cm^−1^ band ratios are omitted due to the overlap of the 19800 cm^−1^ band with the 21000 and 22000 Cr^6+^ bands. C) shows the intensity ratio of deconvoluted UV‐Vis‐NIR DRS bands of the Cr^6+^_monochromate_ band at 27000 cm^−1^ to the Cr^6+^_dichromate_ band at 31000 cm^−1^ before and after reduction with TEB or TEAl.

Consequentially, Figure 6 shows how Cr_reduced_/Cr^6+^_dichromate_ band intensity ratios develop as a function of increasing TEB (6A) and TEAl (6B) amounts, with Cr_reduced_ being reflected by the intensity at the maxima found for the deconvoluted bands. Lastly, Figure 6C shows the development of the Cr^6+^_monochromate_ (27000 cm^−1^)/Cr^6+^_dichromate_ (31000 cm^−1^) ratio as a function of type and amount of co‐catalyst.^13^, ^14^, ^64^

(1) $$\frac{I\;\left({11900\;cm}^{-1}\right)}{I\;\left({31000\;cm}^{-1}\right)}=\frac{\epsilon_{reduced}{\left[C\right]}_{reduced}}{\epsilon_{chromate}{\left[C\right]}_{chromate}}=K\;\frac{{\left[C\right]}_{reduced}}{{\left[C\right]}_{chromate}}$$

As discussed, larger amounts of TEB appeared to affect the reduction chemistry only to a small extent. However, Figure 6A demonstrates an inverse relationship between relative amounts of Cr^2+^ (11900/31000 cm^−1^) and Cr^3+^ (16000/31000 cm^−1^) with increasing amounts of TEB. Furthermore, Figure 6C demonstrates that predominantly the mono‐chromates react with TEB, where the ratio appeared to be barely affected by the added amount. On basis of these findings, it is likely that larger amounts of TEB enhance the formation of Cr^3+^ at the expense of Cr^2+^, which offers an explanation for, e. g., decreased catalyst activities of larger amounts: in turn rationalized by deactivation/destruction of otherwise active sites.

The more significant reduction with more TEAl was already discussed and is further illustrated in Figure 6B, where the increase of the 11100 cm^−1^ band is explained by increasing amounts of two types of mono‐grafted mono‐alkyl Cr^2+^ sites and the increase of the 15200 cm^−1^ is explained by an increase of inaccessible Cr^3+^ sites. It is likely that the increased formation of these species is due to overreduction of the bis‐grafted bis‐alkyl Cr^4+^ sites contributing at 19800 cm^−1^. Interestingly, Figures S12, S14 and S16 do demonstrate increasing intensities for the band at 19800 cm^−1^, inferring relatively larger amounts of bis‐grafted bis‐alkyl Cr^4+^ sites, related to TEAl exploiting additional Cr^6+^ sites in the favor of active site formation. However, the bands do not increase thrice/twice in intensity if the added TEAl amount is tripled (1.5→5.0 mol. eq.) or doubled (5.0→10.0 mol. eq.). This is further supported by Figure 6C where a small, albeit continual, decrease of the 27000/31000 cm^−1^ ratio is observed.

## 3 Conclusions

In this work we found that careful selection of the type and amount of co‐catalyst allows for uniquely tailoring Cr/SiO_2_ ethylene polymerization, with tunable catalyst activities, induction periods and polyethylene characteristics.

Semi‐batch ethylene polymerization experiments revealed that the added co‐catalysts significantly enhanced the catalytic performance. TEB raised the catalyst activity to a larger extent, however, suffered from decreased catalyst performances at increased amounts. On the other hand, TEAl increased the catalyst activity to a smaller extent, but did not yet suffer catalyst deactivation for the used amounts, suggesting we are working under the deactivation/destruction treshold. Addition of either co‐catalyst greatly benefited the reduction of the induction period, in both instances a minimum of 8 min was attained.

Addition of the different types and amounts of co‐catalyst materials allowed for finely tailoring the polymer product characteristics centered around a HDPE with a density of 956 kg/m^3^ which was produced in the absence of a co‐catalyst. Addition of TEAl resulted in the production of HDPE with a decreased density, while TEB, on the other hand, produced HDPE with increased densities.

The UV‐Vis‐NIR DRS experiments revealed that TEB and TEAl distinctly affected the reduction chemistry of the Cr/SiO_2_ Phillips catalyst. TEB primarily reduced Cr^6+^ to Cr^2+^_Oh/Td_ and Cr^3+^_Oh_, producing an active ethylene polymerization catalyst. Increased amounts of TEB resulted in the formation of Cr^3+^_Oh_, at the expense of Cr^2+^_Oh/Td_, indicating the potential of TEB to deactivate the catalyst while simultaneously inferring that the 11000 cm^−1^ Cr^2+^ band participates in ethylene polymerization.

The effect of TEAl was highly dependent on the added amount. With small amounts of TEAl producing bis‐grafted bis‐alkyl Cr^4+^ species and only minor amounts of Cr^2+^ and Cr^3+^, while simultaneously enhancing the reducibility by ethylene. Increased amounts of TEAl raised the reduction of Cr^6+^ into bis‐grafted bis‐alkyl Cr^4+^ species but also coincided with overreduction of these Cr^4+^ species to Cr^2+^ and Cr^3+^ species.

If anything, our results clearly confirm that the type and amount of co‐catalyst is pivotal for controlling the Cr reduction chemistries and consequential active site formation. In addition, the results indicate that the co‐catalyst remains a significant contributor to catalyst performances and bulk properties, even after the initial reduction stage.

## Experimental Section

### Sample Preparation

The catalyst samples were provided by SABIC Geleen, the Netherlands. It is a silica Cr‐catalyst with a ∼1.0 wt% Cr loading, a surface area of 625 m^2^/g, a pore volume of 2.41 mL/g and a D50 particle size distribution of 52.8 μm. The catalyst was calcined at 650 °C via a SABIC‐proprietary technique to yield the active CrO_x_/SiO_2_ catalyst.

### Batch‐Reactor Catalyst Testing

Slurry phase polymerization reactions with a Cr/SiO_2_ Phillips‐type catalyst were performed in a 5 L semi‐batch reactor at SABIC Geleen, during which induction time, catalyst yield and total polymerization time were monitored. The batch reactor was loaded with 1071 g isobutane and heated to 99 °C. 830 mg H_2_ was added and subsequently the reactor was pressurized to 34 bar with C_2_H_4_. The diluent was loaded with 12 mol% C_2_H_4_ and 1 mol% H_2_. Upon reaching the reaction conditions the co‐catalyst was injected with 120 g of isobutane and subsequently the Cr/SiO_2_ catalyst was injected with 180 g of isobutane. Ethylene was fed to the reactor to maintain constant pressure. A catalyst yield of approximately 2700 grams of polyethylene per gram of catalyst was used as an end‐point of the reaction.

### Density Measurements of the Polyethylene Products

The polymer Melt Flow Indices were measured at 190 °C on a Zwick/Roell 4106 Extrusion Plastometer. Polymer amounts of 3.5 and 2.8 g were used for respectively measuring the MFI 21.6 and MFI 5.0, Polymer densities were measured following the standardized ISO 1183 procedure.

### GPC‐SEC‐DV‐IR Measurements

Gel permeation chromatography – Size exclusion chromatography – Differential Viscometry – Infrared (GPC‐SEC‐DV‐IR) was carried out on a PolymerChar GPC‐IR system running at 160 °C equipped with a Polymer Char IR5 infrared detector and a PolymerChar viscometer. The column set consisted of three Polymer Laboratories 13 um PLgel Olexis 300 × 7.5 mm columns. PE molar mass calibration was performed with linear PE standards in the range of 0.5–2800 kg/mol (Mw/Mn=4 to 15).

### UV‐Vis‐NIR Diffuse Reflectance Spectroscopy

The in‐situ UV‐VIS‐NIR Diffuse Reflectance Spectroscopy (UV‐Vis‐NIR DRS) measurements were performed on a Varian Cary 500 Scan spectrophotometer with a DRS accessory. Measurements were performed in the spectroscopic range of 4000–45000 cm^−1^ with 33 ms data point scan time and spectroscopic resolution of 17 cm^−1^ and 7 cm^−1^ in respectively the 12500–45000 cm^−1^ and 4000–12500 cm^−1^ spectroscopic range. Two artefacts in the measured spectra were corrected for the detector/grating and light source changeovers at 12500 cm^−1^ and 28570 cm^−1^, while the spectroscopic feature appearing at 11250 cm^−1^ is due to an instrumental artefact. For every measurement, the cell was loaded in a N_2_ glovebox, preventing the samples from contact with atmospheric oxygen and water. The samples were measured against a Teflon‐white measured in the same cell loaded with the same volume of 30 μm beads of Teflon powder. For measuring the catalyst materials, 100 mg of catalyst material was loaded in the specially designed cell. Subsequently, the desired volume of metal‐alkyl co‐catalyst was injected via the septum into the N_2_ gas stream, tri‐ethyl borane (Sigma‐Aldrich, 1 M in Hexanes), or a solution of 1 M tri‐ethyl aluminum (Sigma‐Aldrich, 93 %) in hexanes (dried over 4 Å molsieves). After addition of the co‐catalyst, spectra were recorded every 3 min for 15 min. Subsequently, the N_2_ gas stream of 10 mL/min was switched to a gas stream of 10 mL/min C_2_H_4_. The system was heated to 150 °C with 5 °C/min, spectra were recorded at 40 °C, 60 °C, 80 °C, 100 °C, 120 °C and 150 °C.

## Conflict of interest

The authors declare no conflict of interest.

## Supporting information

As a service to our authors and readers, this journal provides supporting information supplied by the authors. Such materials are peer reviewed and may be re‐organized for online delivery, but are not copy‐edited or typeset. Technical support issues arising from supporting information (other than missing files) should be addressed to the authors.

Supplementary
