# Supplementary material for: Tuning the Redox Chemistry of a Cr/SiO2 Phillips Catalyst for Controlling Activity, Induction Period and Polymer Properties
Source: Chemphyschem. 2020 Jul 6;21(15):1665–74. doi: 10.1002/cphc.202000488 (PMC7496818; doi:10.1002/cphc.202000488)
Supplement: Supplementary file 1 — Supplementary [file CPHC-21-1665-s001.pdf]

ChemPhysChem

Supporting Information

Tuning the Redox Chemistry of a Cr/SiO_2 Phillips Catalyst for Controlling Activity, Induction Period and Polymer Properties

Maarten K. Jongkind, Theo van Kessel, Marjolein E. Z. Velthoen, Nic. Friederichs, and Bert M. Weckhuysen*

Supporting Information

S1. Semi Batch Ethylene-Polymerization Reactions

Table S1. The applied reaction conditions for the performed semi batch-ethylene polymerization reactions. During these reactions 1371 g of isobutane was used as a diluent, all the reactions required ~165 mg CrO_x/SiO_2 catalyst. 830 mg of H_2 was added for ensuring a 1 mol% H_2 concentration in the diluent, also the diluent was saturated with 12 mol% C_2H_2. Anti-fouling agent was simultaneously injected with the catalyst material.

Temp (˚C)	[C2] (mol%)	[H2] (mol%)	[AFA] (ppm)	Cat (mg)	Isobutane (g)
99	12	1.0	2.0	~165	1051

The 1071g isobutane was added to a 5L CSTR reactor and heated to 99°C At 99°C 830 mg of H_2 was added to the reactor to reach 1,0 mol% H_2 in the liquid phase. After the addition of the H_2, the reactor was pressured up to 34 bar with ethylene in order to reach 12 mol% C_2H_4 in the diluent. The required amount of cocatalyst was injected with 120 g of isobutane while ethylene was used to keep the pressure in the reactor at 34 bar. The catalyst was injected with 180 g of isobutane and anti-fouling agent (AFA). Ethylene was continuously added to keep the pressure at 34 bar. After the catalyst was injected the ethylene totalizer was reset to zero and the polymerization time started. The time of catalyst injection was marked as t=0 and the induction period was defined as the time from t=0 until the moment at which ethylene had to be actively fed to the reactor to maintain the reactor pressure of 34 bar.

It is worth noting that the Melt Flow Index Ratio ($MFI_{21.6}/MFI_5$) gives an indication of the rheological broadness of the material. This broadness is affected by the molecular weight distribution (Mw/Mn = MWD) and the presence of long-chain branching (LCB). Generally, increasing values for the melt-flow index ratio indicate a broader MWD and/or more LCB.[1]

The Powder Bulk Density (see Tables **S2** and **S3**) is the mass of the product powder per volume unit. It is a measure for the efficiency with which polymer particles can pack. It is, amongst others, dependent on the particle shape, particle size distribution and particle compactness. Higher Powder Bulk Densities generally indicate that more product is contained per volume unit, which is a desirable feature for optimizing the amount of polyethylene produced per reactor volume. Broader Particle Size distributions in combination with increased spans can indicate fragmentation of the growing polymer particle.

Table S2. Catalyst activity and product properties of the performed ethylene polymerization reactions in the presence of an increasing concentration of tri-ethyl borane (TEB). The Induction Time was the time ranging from the moment of catalyst injection to the moment of maintaining the reactor pressure by ethylene addition. The polymerization time is defined as the time it took to reach a Catalyst Yield of approximately 2700 kg/kg. The MFI 5 and MFI 21.6 Melt Flow Indices were measured according to the ISO 1183 method. D10, D50 and D90 Particle Size Distributions were measured and from ((D90-D10)/D50) the particle span was calculated.

TEB-Concentration (ppm)	Induction Time (min)	Polymerization Time (min)	Catalyst Yield ($g_{PE}g_{cat}^{-1}$)	MFI 5 (dg/min)	MFI 21.6 (dg/min)	MFIR	PBD (kg/m^3)	PSE D10 (µm)	PSE D50 (µm)	PSE D90 (µm)	Span (D90-D10)/D50
**0.0**	**28**	**169**	**2649**	**0.13**	**2.7**	**19.8**	**442**	**336**	**636**	**889**	**0.87**
0.0	46	197	2786	0.13	2.6	20.8	378				
**0.05**	**9**	**69**	**2744**	**0.11**	**3.1**	**28.2**	**347**	**239**	**550**	**811**	**1.04**
0.05	9	55	2697	0.17	4.8	28.2	286	248	545	793	1.00
0.10	5	45	3174	0.15	5.0	33.3	310	246	535	801	1.04
0.10	9	51	2933	0.18	5.3	29.4	290	252	531	785	1.00
0.15	10	54	2686	0.19	5.3	27.9	285	255	534	786	1.00
**0.15**	**8**	**48**	**2676**	**0.20**	**5.8**	**29.0**	**280**	**227**	**478**	**747**	**1.09**
0.20	8	47	2626	0.22	7.0	31.8	290	229	484	745	1.07
0.20	6	39	2638	0.23	7.5	32.6	279	220	488	763	1.11
0.25	7	34	2793	0.26	8.2	31.6	274				
0.25	6	34	2815	0.29	9.2	31.7	256	222	467	741	1.11
0.30	6	38	1789	0.26	7.9	30.4	276	229	482	741	1.06
**0.30**	**5**	**34**	**2712**	**0.26**	**8.5**	**32.7**	**268**	**216**	**464**	**728**	**1.10**
0.35	4	30	2824	0.33	9.8	29.6	266	210	462	747	1.16
0.35	5	32	2717	0.30	10.7	35.7	251	221	458	724	1.10
0.70	4	32	2828	0.35	10.4	29.7	250	217	478	736	1.09
0.70	5	34	2793	0.35	10.9	31.1	241	235	510	776	1.06
1.50	5	39	2740	0.43	12.9	30.0	260	238	530	783	1.03
**1.50**	**3**	**39**	**2714**	**0.43**	**12.4**	**28.8**	**254**	**203**	**504**	**766**	**1.12**
3.00	4	43	2681	0.38	11.9	31.3	269	239	548	841	1.10
**3.00**	**6**	**47**	**2747**	**0.32**	**10.9**	**34.3**	**271**	**241**	**582**	**802**	**0.96**

Table S3. Catalyst activity and product properties of the performed ethylene polymerization reactions in the presence of an increasing concentration of tri-ethyl aluminum (TEAl). The Induction Time was the time ranging from the moment of catalyst injection to the moment of maintaining the reactor pressure by ethylene addition. The polymerization time is defined as the time it took to reach a Catalyst Yield of approximately 2700 kg/kg. The MFI 5 and MFI 21.6 Melt Flow Indices were measured according to the ISO 1183 method. D10, D50 and D90 Particle Size Distributions were measured and from ((D90-D10)/D50) the particle span was calculated.

TEAl-Concentration (ppm)	Induction Time (min)	Polymerization Time (min)	Catalyst Yield ($g_{PE}g_{cat}^{-1}$)	MFI 5 (dg/min)	MFI 21.6 (dg/min)	MFIR	PBD (kg/m^3)	PSE D10 (µm)	PSE D50 (µm)	PSE D90 (µm)	Span (D90-D10)/D50
**0.0**	**28**	**169**	**2649**	**0.13**	**2.7**	**19.8**	**442**	**336**	**636**	**889**	**0.87**
0.0	46	197	2786	0.13	2.6	20.8	378				
0.05	13	86	2707	0.10	2.5	25.0	509	189	493	760	1.16
0.05	18	98	2882	0.09	2.3	25.6	509	177	502	770	1.18
0.10	18	99	2606	0.11	2.3	20.9	510	211	553	872	1.19
0.10	21	113	2725	0.11	2.3	20.9	524	226	555	859	1.14
0.15	8	75	2782	0.11	2.8	25.5	444	130	446	745	1.38
0.15	9	70	2946	0.12	2.9	24.2	412	107	384	703	1.55
0.20	8	63	2769	0.12	3.2	26.7	420	167	513	816	1.27
0.20	7	68	2716	0.11	2.9	26.4	450	115	397	701	1.48
0.25	8	71	2695	0.12	2.8	23.3	405	186	497	766	1.17
0.25	6	66	2675	0.15	3.6	24.0	394	131	437	735	1.38
0.30	7	68	2719	0.13	3.2	24.6	410				
**0.30**	**7**	**62**	**2751**	**0.13**	**3.3**	**25.4**	**373**	**156**	**490**	**785**	**1.28**
0.35	5	60	2723	0.15	3.7	24.7	376	142	463	716	1.24
0.35	5	66	2682	0.13	3.2	24.6	391	189	551	887	1.27
0.70	6	58	3012	0.15	3.9	26.0	362	173	549	857	1.25
0.70	7	58	2705	0.15	4.0	26.7	358	179	516	782	1.17
**1.50**	**5**	**50**	**2673**	**0.25**	**6.5**	**26.0**	**380**	**165**	**472**	**750**	**1.24**
1.50	6	51	2663	0.23	6.2	27.0	389	152	425	682	1.25
**3.00**	**7**	**53**	**2770**	**0.32**	**7.7**	**24.1**	**362**	**161**	**401**	**685**	**1.31**
3.00	9	55	2677	0.31	7.4	23.9	359	163	411	700	1.30

Table S4. An overview of the Polymer Densities for the experiments with 0.30, 1.50 and 3.00 ppm of TEB and TEAl respectively. Furthermore, molecular structure paramaters, in terms of Mn, Mw, Mz and Mw/Mn were investigated with GPC-SEC-DV-IR and are presented.

Exp.	Polymer Density (kg/m^3)	Mn (kDa)	Mw (kDa)	Mz (kDa)	Mw/Mn
	956.1	19	360	2600	18.6
**TEB**					
0.30	960.0	11	330	3000	30.3
1.50	958.8	11	220	1700	20.8
3.00	958.9	9	270	2400	29.1
**TEAl**					
0.30	954.6	16	370	2900	23.6
1.50	953.4	12	340	2900	29.3
3.00	953.5	12	320	2900	28.0

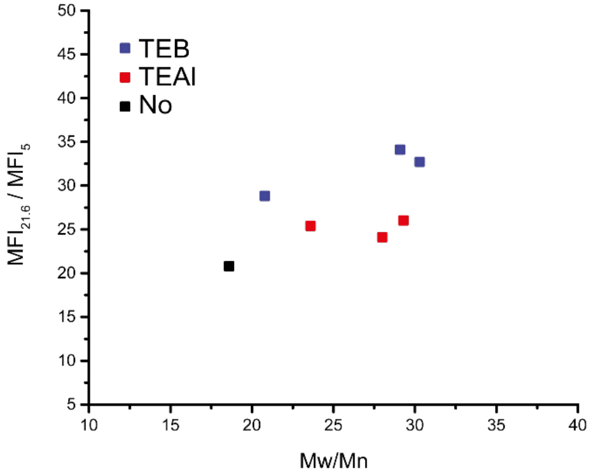

Figure S1. MFIR plotted versus the Mw/Mn for the materials from Table **S4** after analysis of the Gel Permeation Chromatography – Size Exclusion chromatography – Differential Viscometry – Infrared measurements. Higher values for the MFIR at a specific Mw/Mn can indicate LCB.

S2. UV-Vis-NIR Diffuse Reflectance Spectroscopy (DRS) experimental procedure and results

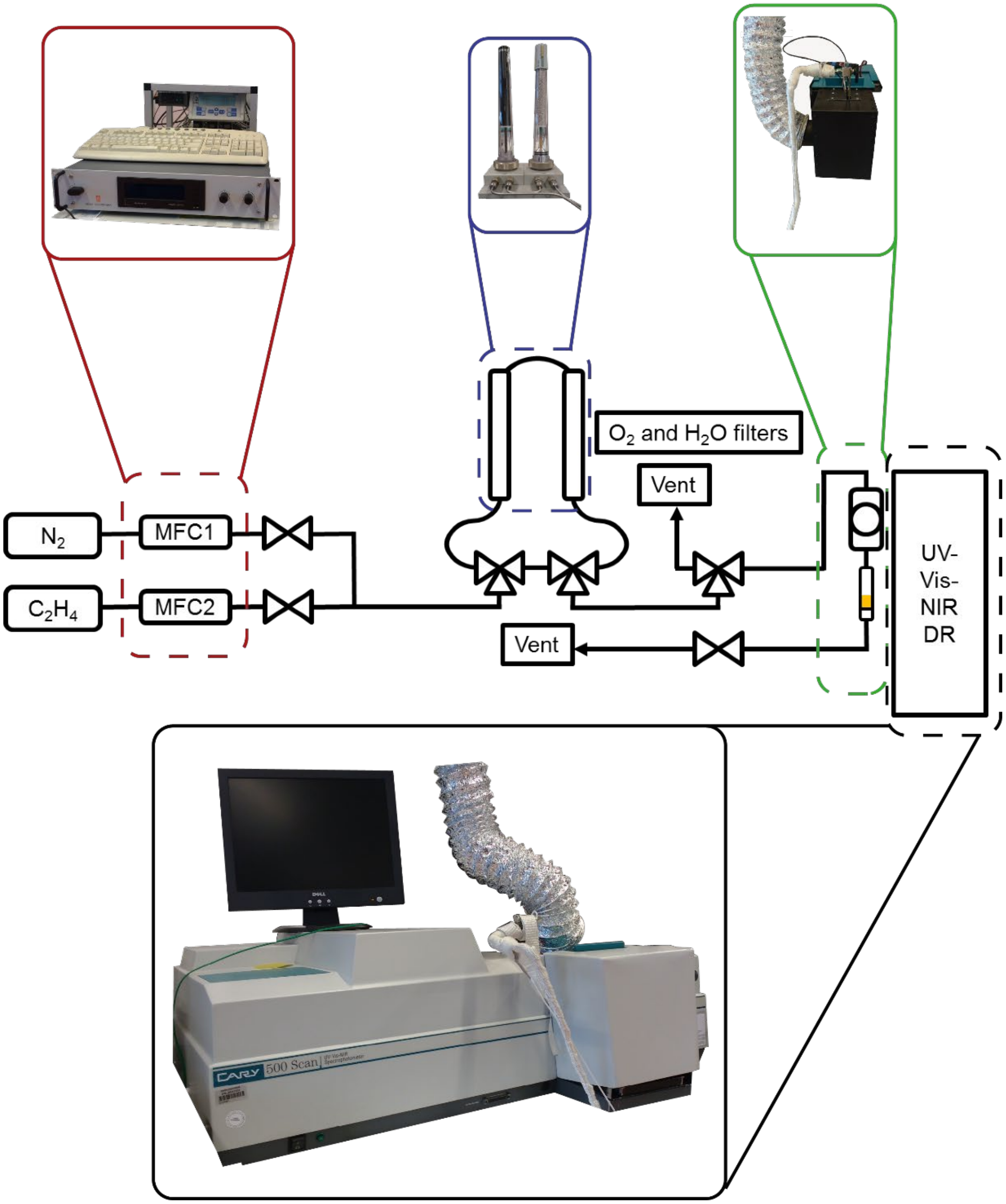

Scheme S1. Overview of the *in-house* UV-Vis-NIR DRS setup, with the possibility of feeding respectively ethylene or nitrogen. The volume of gas per minute could separately be regulated with two mass flow controllers. After which they passed another two-way valve and the lines joined. Subsequently impurities were scrubbed from the gas-feed by O_2 and H_2O filters, for circumventing contact of the catalyst material with atmospheric H_2O and O_2. After the two columns, the line passed a three-way valve either to a vent or to the catalyst material. The catalyst material was placed in a specially designed quartz cell on a frit, in each experiment 100mg catalyst was used. Directly above the catalyst bed was a septum via which the desired volume of co-catalyst could be injected.

Scheme **S1** is an overview of the used set-up for our *in-situ* UV-Vis-NIR DRS experiments. Here, we had the possibility of using two gases: nitrogen or ethylene as well as a combination of the two. Both gasses passed a Mass-Flow Controller, O_2 and H_2O filters before reaching a three-way valve, via which the gas could be directed to the ventilation or to the cell. The catalyst material was loaded in a Quartz cell, which could be aligned on the UV-Vis-NIR DRS spectrometer. An injection module for injection of the co-catalyst was placed directly above the catalyst bed.

Figure **S2** shows the experiment in which ethylene was used as reductant. The reactor was heated to 150 ˚C before switching the gas stream to ethylene. Three key-spectra are shown here: 1) pristine catalyst material of this experiment 2) ethylene reduced catalyst material and 3) spectrum after polymerization. Addition of the different co-catalysts resulted in different reduction pathways, which are discussed in the main manuscript. Tables **S5**, **S6** and **S7** give an overview of the used deconvolution parameters for the experiments performed with the different reductants, respectively ethylene and CO (Table **S5**), TEB (Table **S6**) and TEAl (Table **S7**).

Table S5. An overview of the used deconvolution parameters for the UV-Vis-NIR DRS spectra of the Cr^{6+}/SiO_2 catalyst before reduction and also after reduction in 50 v/v% N_2/ethylene at 150 °C and ambient pressure as well as after reduction by CO at 350 °C and ambient pressure.

Material	Bands (HWHM)	Comments
Cr^{6+}/SiO_2	15000 (3000), 21700 (3000), 33900 (3000)	Cr^{3+}_{Oh}
	20700 (1400), 26300 (2800), 34000 (2000), 37200 (2500)	$Cr^{6+}_{monochromate}$
	22200 (1600), 31100 (3000), 35000 (2000), 40100 (3000)	$Cr^{6+}_{dichromate}$
	46000 (3500)	SiO_2 support
**Ethylene Reduced**	9500 (1500), 13900 (2800), 19300 (2000), 24600 (2500)	$Cr^{2+}_{Oh/Td}$ Oh = Pseudo-octahedral
	15500 (3000), 21500 (3000), 33900 (3000)	Cr^{3+}_{Oh}
	20700 (1400), 26400 (2800), 34000 (2000), 37300 (2500)	$Cr^{6+}_{monochromate}$
	22400 (1600), 31000 (3000), 35030 (2000), 40400 (3000)	$Cr^{6+}_{dichromate}$
	46200 (3500)	SiO_2 support
**CO Reduced**	7800 (2000)	Cr^{2+}_{Td}
	12000 (2200)	Cr^{2+}_{Oh}
	15700 (2800), 21500 (2800), 32700 (2800)	Cr^{3+}_{Oh}
	28500 (3400), 37500 (4200)	Cr^{2+} CT
	46500 (4000)	SiO_2 Support

Table S6. An overview of the used parameters for the each of the spectral deconvolutions of the UV-Vis-NIR DRS spectra obtained from the experiments with TEB as the co-catalyst. The parameters are provided for the deconvoluted Cr^{6+}/SiO_2 UV-Vis-NIR DRS spectra before reduction by TEB and 15 min after pre-contacting the material with TEB.

Material	Bands (HWHM)	Comments
**TEB**		
Cr^{6+}/SiO_2	15000 (3000), 21500 (3000), 33900 (3000)	Cr^{3+}_{Oh}
	20700 (1400), 26400 (2800), 34000 (2000), 37600 (2500)	$Cr^{6+}_{monochromate}$
	22000 (1600), 31000 (3000), 35000 (2000), 40000 (3000)	$Cr^{6+}_{dichromate}$
	45900 (3500)	SiO_2 support
1.50 B:Cr Mole Ratio	9400 (1400), 11900 (2000), 24000 (1500)	$Cr^{2+}_{Oh/Td}$ Oh = Pseudo-octahedral
	16000 (3000), 21500 (3000), 33900 (3000)	Cr^{3+}_{Oh}
	21000 (1400), 26900 (2800), 34000 (2000), 37000 (2500)	$Cr^{6+}_{monochromate}$
	22300 (1600), 31100 (3000), 35000 (2000), 40200 (3000)	$Cr^{6+}_{dichromate}$
	46400 (3500)	SiO_2 support
Cr^{6+}/SiO_2	15100 (3000), 21500 (3000), 33900 (3000)	Cr^{3+}_{Oh}
	20700 (1425), 26400 (2800), 34000 (2000), 37600 (2500)	$Cr^{6+}_{monochromate}$
	22100 (1600), 31000 (3000), 35000 (2000), 40200 (3000)	$Cr^{6+}_{dichromate}$
	46000 (3500)	SiO_2 support
5.0 B:Cr Mole Ratio	9400 (1400), 11900 (2000), 24000 (1500)	$Cr^{2+}_{Oh/Td}$ Oh = Pseudo-octahedral
	15900 (3000), 21500 (3000), 33900 (3000)	Cr^{3+}_{Oh}
	21000 (1400), 27200 (2800), 34100 (2000), 37300 (2800)	$Cr^{6+}_{monochromate}$
	22400 (1600), 31400 (3000), 35100 (2000), 40600 (3000)	$Cr^{6+}_{dichromate}$
	45800 (3500)	SiO_2 support
Cr^{6+}/SiO_2	15100 (3000), 21500 (3000), 33900 (3000)	Cr^{3+}_{Oh}
	20600 (1400), 26300 (2800), 34000 (2000), 37600 (2500)	$Cr^{6+}_{monochromate}$
	22100 (1800), 31000 (2850), 35500 (2000), 40600 (3000)	$Cr^{6+}_{dichromate}$
	46600 (3500)	SiO_2 support
10.0 B:Cr Mole Ratio	9400 (1400), 11900 (2000), 24000 (1500)	$Cr^{2+}_{Oh/Td}$ Oh = Pseudo-octahedral
	16000 (3000), 21500 (3000), 33500 (3000)	Cr^{3+}_{Oh}
	21000 (1410), 26900 (2800), 34000 (2000), 37000 (2800)	$Cr^{6+}_{monochromate}$
	22300 (1600), 31100 (3000), 35000 (2000), 40200 (3000)	$Cr^{6+}_{dichromate}$
	46400 (3500)	SiO_2 support

Table S7. An overview of the used parameters for the each of the spectral deconvolutions of the UV-Vis-NIR DRS spectra obtained from the experiments with TEAl as the co-catalyst. The parameters are provided for the deconvoluted Cr^{6+}/SiO_2 UV-Vis-NIR DRS spectra before reduction by TEAl and 15 min after pre-contacting the material with TEAl.

Material	Bands (HWHM)	Comments
**TEAl**		
Cr^{6+}/SiO_2	15000 (3000), 21600 (3000), 33900 (3000)	Cr^{3+}_{Oh}
	20700 (1400), 26300 (2800), 33900 (2000), 37100 (2500)	$Cr^{6+}_{monochromate}$
	22000 (1600), 31000 (3000), 35000 (2000), 40200 (3000)	$Cr^{6+}_{dichromate}$
	46000 (3500)	SiO_2 support
1.50 Al:Cr Mole Ratio	11500 (2500), 15200 (2500), 19800 (1500), 22800 (1120)	$Cr^{2+}_{Oh/Td\ Oh\ =\ Pseudo\text{-}octahedral}$ / $Cr^{3+}_{reduced}$ / Cr^{4+}_{red}
	16000 (3000), 21500 (3000), 33900 (3000)	Cr^{3+}_{Oh}
	20800 (1400), 26600 (2800), 34000 (2000), 37000 (2750)	$Cr^{6+}_{monochromate}$
	22000 (1600), 30900 (3000), 35000 (2000), 39500 (3000)	$Cr^{6+}_{dichromate}$
	45600 (3500)	SiO_2 support
Cr^{6+}/SiO_2	15000 (3000), 21500 (3000), 33900 (3000)	Cr^{3+}_{Oh}
	20700 (1400), 26400 (2800), 34000 (2000), 37300 (2500)	$Cr^{6+}_{monochromate}$
	22000 (1600), 31300 (3000), 35000 (2000), 36900 (3000)	$Cr^{6+}_{dichromate}$
	46600 (3500)	SiO_2 support
5.00 Al:Cr Mole Ratio	11200 (2500), 15200 (2500), 19800 (1500), 24000 (1050)	$Cr^{2+}_{Oh/Td\ Oh\ =\ Pseudo\text{-}octahedral}$ / $Cr^{3+}_{reduced}$ / Cr^{4+}_{red}
	16000 (3000), 21500 (3000), 33900 (3000)	Cr^{3+}_{Oh}
	21000 (1400), 27000 (2800), 34000 (2000), 37000 (2750)	$Cr^{6+}_{monochromate}$
	22100 (1600), 31200 (3000), 35000 (2000), 40000 (3000)	$Cr^{6+}_{dichromate}$
	46400 (3500)	SiO_2 support
Cr^{6+}/SiO_2	15000 (3000), 21500 (3000), 33900 (3000)	Cr^{3+}_{Oh}
	20800 (1400), 26400 (2800), 34000 (2000), 37400 (2500)	$Cr^{6+}_{monochromate}$
	22100 (1680), 31100 (3000), 35000 (2000), 40000 (3000)	$Cr^{6+}_{dichromate}$
	46300 (3500)	SiO_2 support
10.0 Al:Cr Mole Ratio	11300 (2000), 15200 (2500), 19800 (1500), 24000 (1050)	$Cr^{2+}_{Oh/Td\ Oh\ =\ Pseudo\text{-}octahedral}$ / $Cr^{3+}_{reduced}$ / Cr^{4+}_{red}
	16000 (3000), 21500 (3000), 33900 (3000)	Cr^{3+}_{Oh}
	20900 (1400), 26500 (2800), 34000 (2000), 36900 (2750)	$Cr^{6+}_{monochromate}$
	22100 (1600), 31000 (3000), 35300 (2000), 39800 (3000)	$Cr^{6+}_{dichromate}$
	46600 (3500)	SiO_2 support

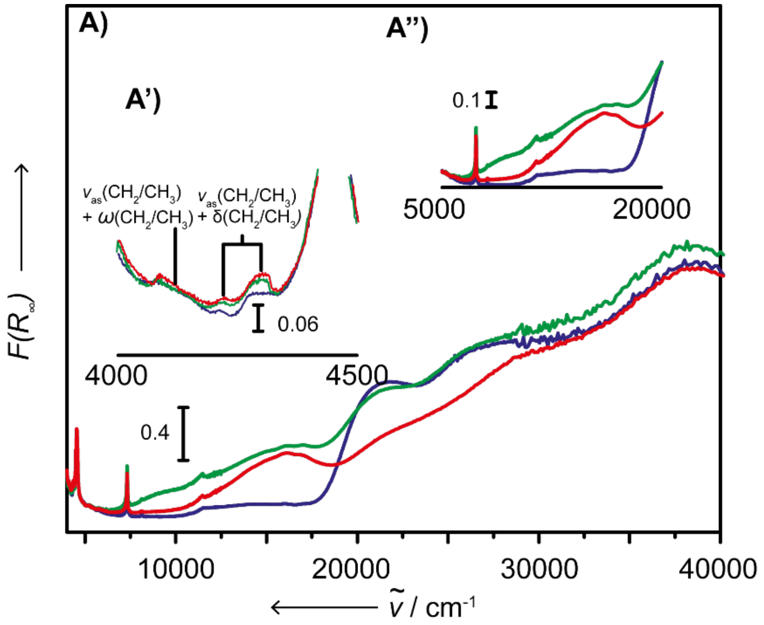

Figure S2. Key spectra from the reduction and polymerization with ethylene at 150 °C and 1 bar. Blue spectrum is the Cr^{6+}/SiO_2 Philips catalyst before reduction, the green spectrum is 20 min after reacting with 10 mL/min ethylene in 10 mL/min N_2 and the red spectrum is 180 min after reacting with 10 mL/min ethylene in 10 mL/min N_2.

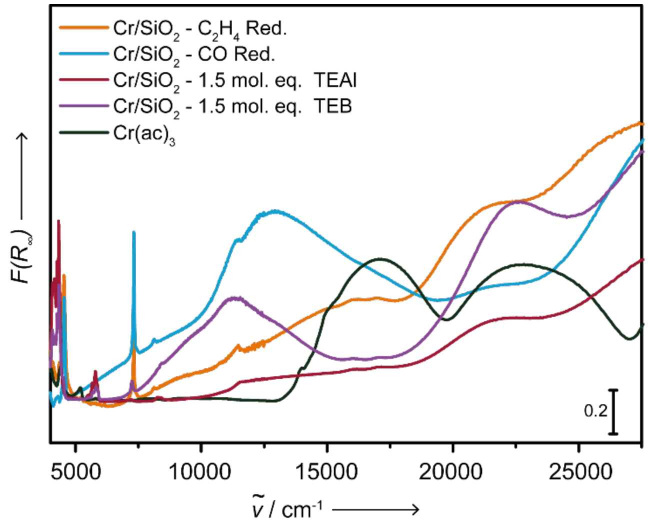

Figure S3. UV-Vis-NIR DRS spectra of the Cr/SiO_2 catalyst 20 min after reduction in a gas mixture of10 mL/min ethylene and 10 mL/min N_2 at 150 °C and 1 bar (orange), by CO at 1 bar and a temperature of 350 ˚C (blue), by 1.50 eq.TEAl at 1 bar in 10 mL/min N_2 (red), by 1.5 eq. TEB at 1 bar in 10 mL/min N_2 (purple) as well as a Cr(III) acetate Cr^{3+}(black) reference material.

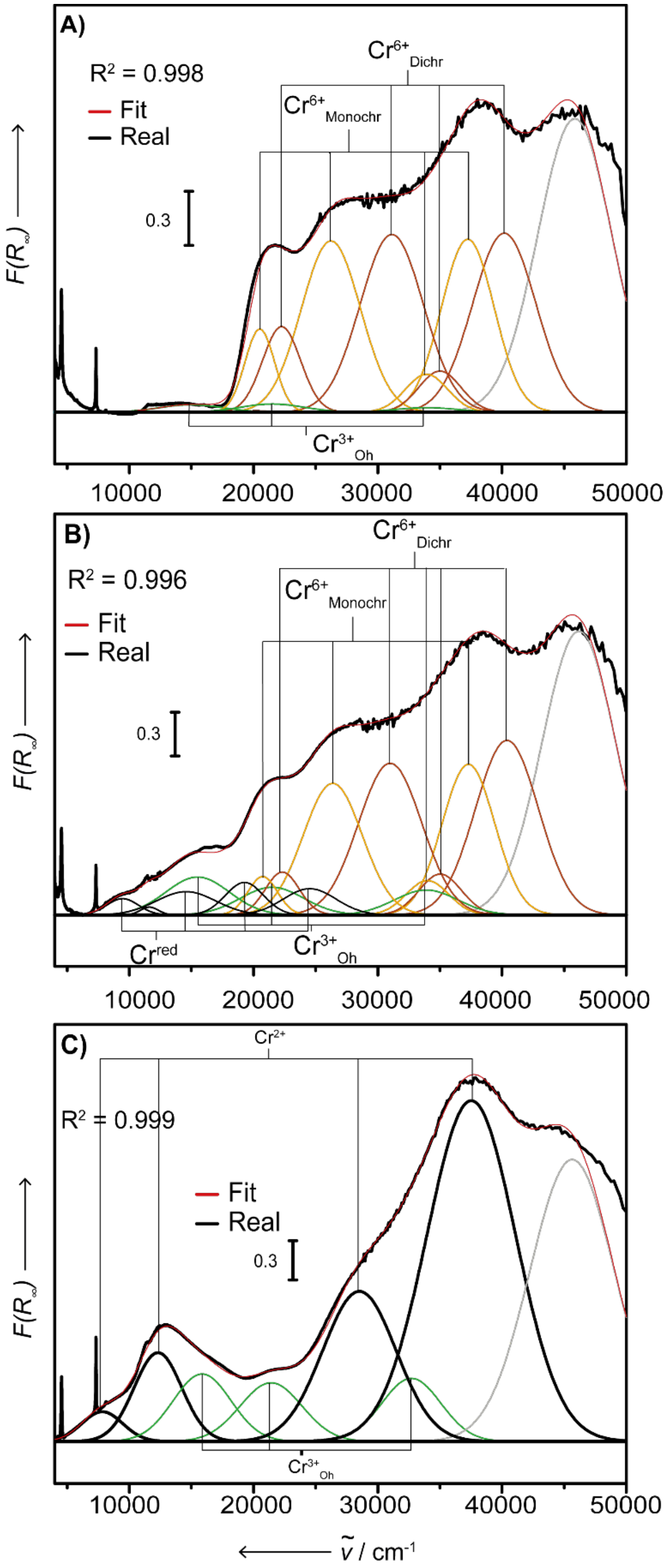

Figure S4. A) Deconvolution of the Cr^{6+}/SiO_2 before reduction by ethylene, at 150 °C and 1 bar nitrogen atmosphere. **B)** Deconvolution of the Cr^{6+}/SiO_2 catalyst 20 min after reduction by 10 mL ethylene in 10 mL N_2 at 150 °C and 1 bar atmosphere. **C)** Deconvolution of the Cr^{6+}/SiO_2 catalyst reduced by CO at 350 °C and 1 bar.

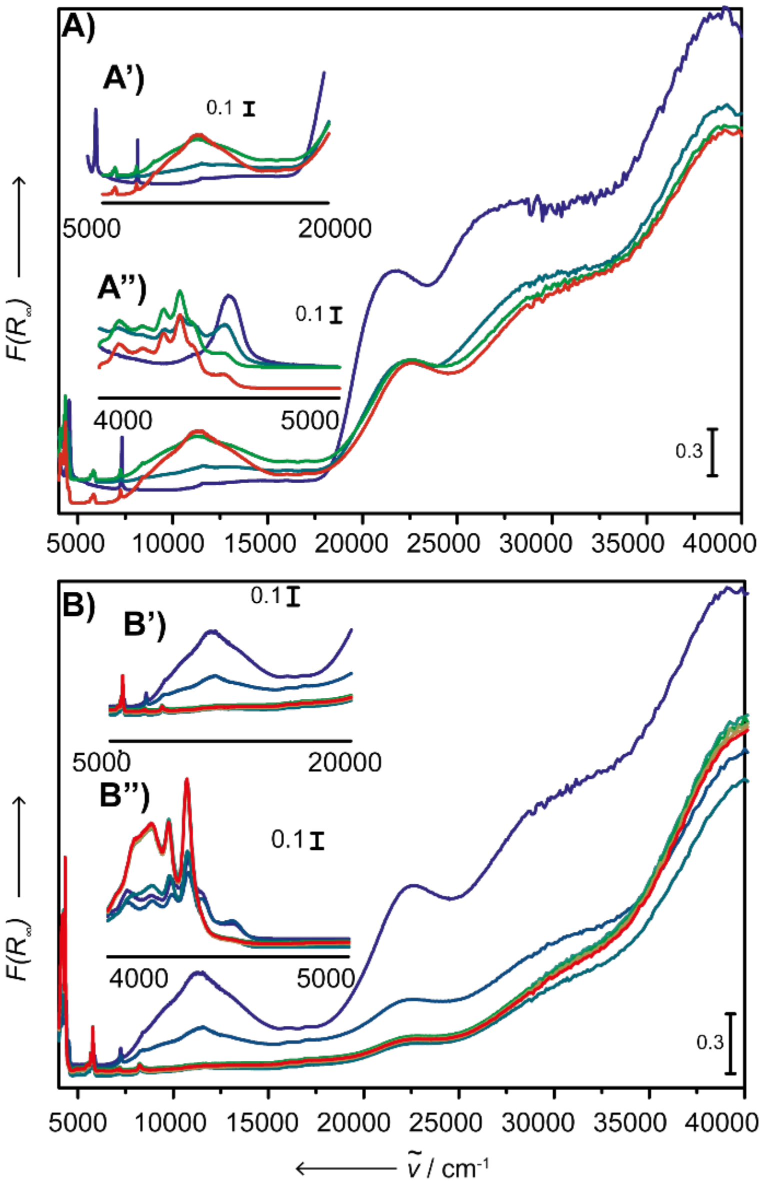

Figure S5. UV-Vis-NIR DRS spectral developments for the experiment with a B:Cr mole ratio of 1.50 from **blue** to **red**, divided in reduction and polymerization. **The spectra, from blue to red, are recorded with *5* min intervals.** **A)** The pre-treatment of the catalyst with TEB under an N_2 stream of 10 mL/min at room temperature and ambient pressure, aiming for a B:Cr mole ratio of 1.50. **B)** Developments after the N_2 stream was switched to a C_2H_4 stream of 10 mL/min, where the spectra were recorded at 20 ˚C intervals.The spectra, from blue to red, are recorded with *5* min intervals.

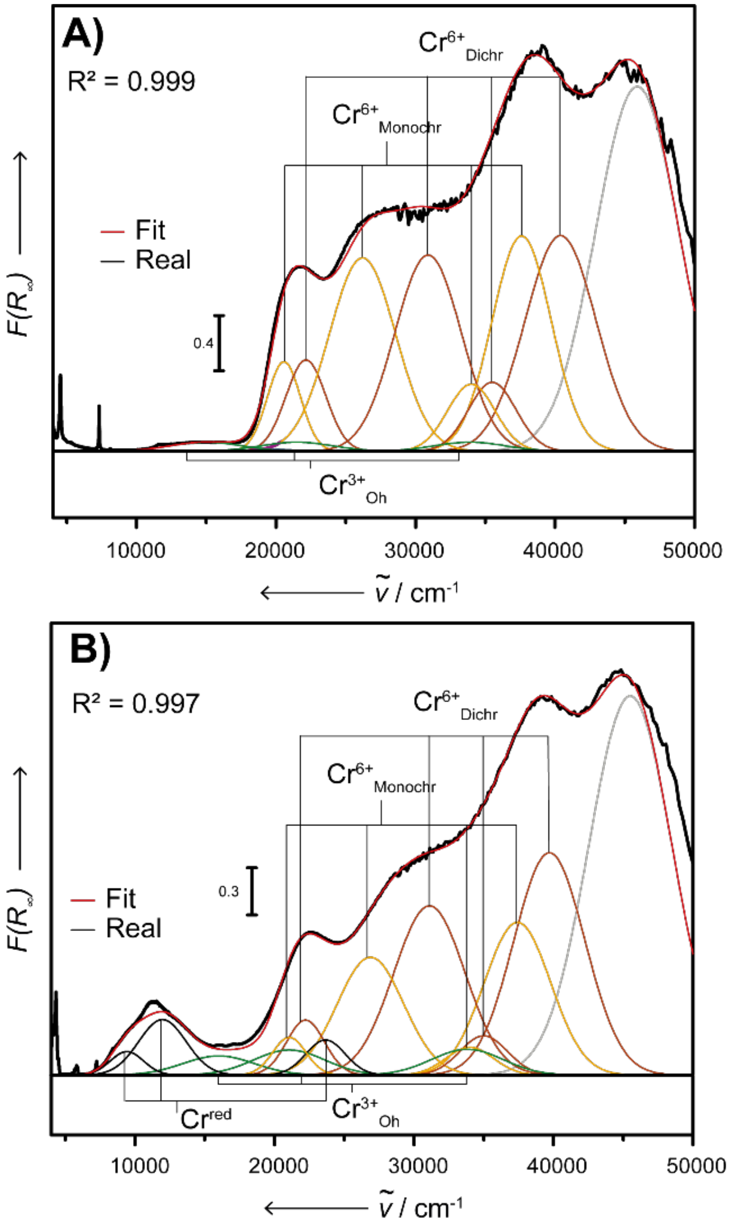

Figure S6. A) Deconvoluted UV-Vis-NIR DRS spectrum of the Cr^{6+}/SiO_2 catalyst material before pre-treatment with 1.50 molecular equivalents of TEB. **B)** Deconvoluted UV-Vis-NIR DRS spectrum after pre-treatment with TEB in a N_2 stream of 10 mL/min, aiming for a B:Cr mole ratio of 1.50. The machinal artefact at 11000 cm^{-1} is not taken into account for deconvolution.

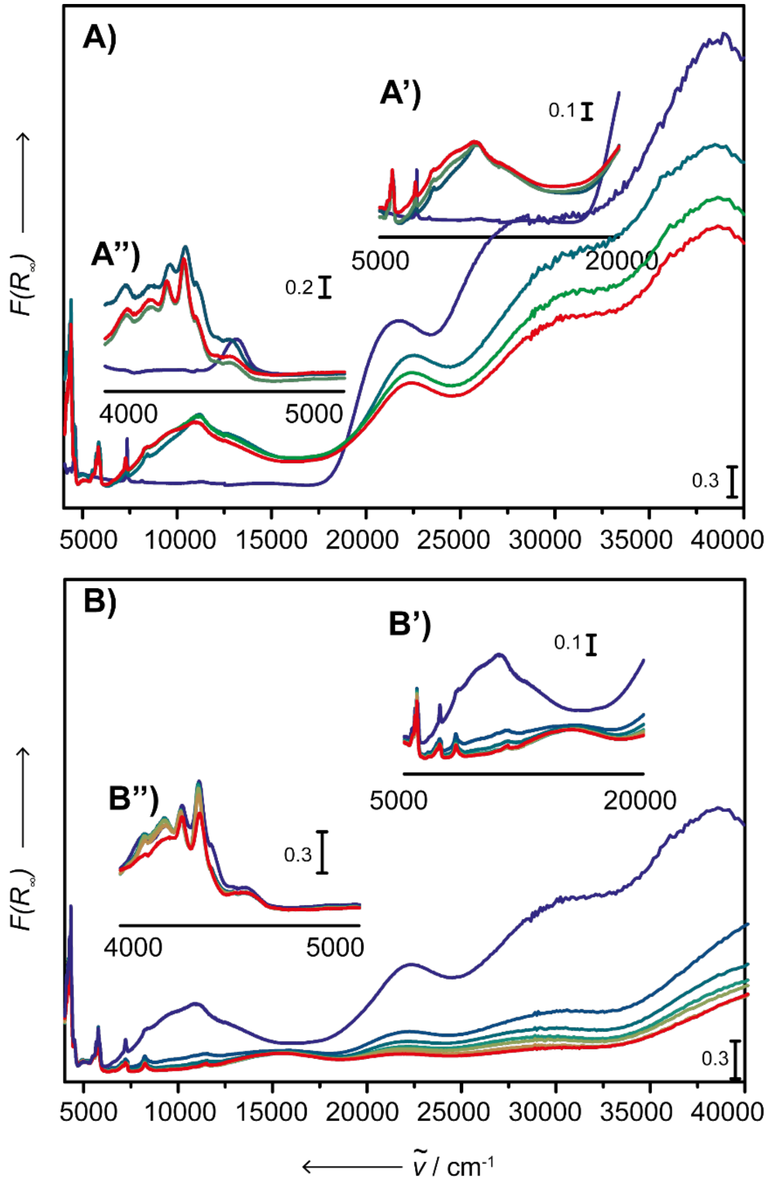

Figure S7. UV-Vis-NIR DRS spectral developments for the experiment with a B:Cr mole ratio of 5.0, from **blue** to **red**, divided in reduction and polymerization. The spectra, from blue to red, are recorded with 5 min intervals. **A)** The pre-treatment of the catalyst with TEB under an N_2 stream of 10 mL/min at room temperature and ambient pressure, aiming for a B:Cr mole ratio of 5.0. **B)** shows spectral developments after the N_2 stream was switched to a C_2H_4 stream of 10 mL/min, where the spectra were recorded at 20 ˚C intervals. The spectra, from blue to red, are recorded with 5 min intervals.

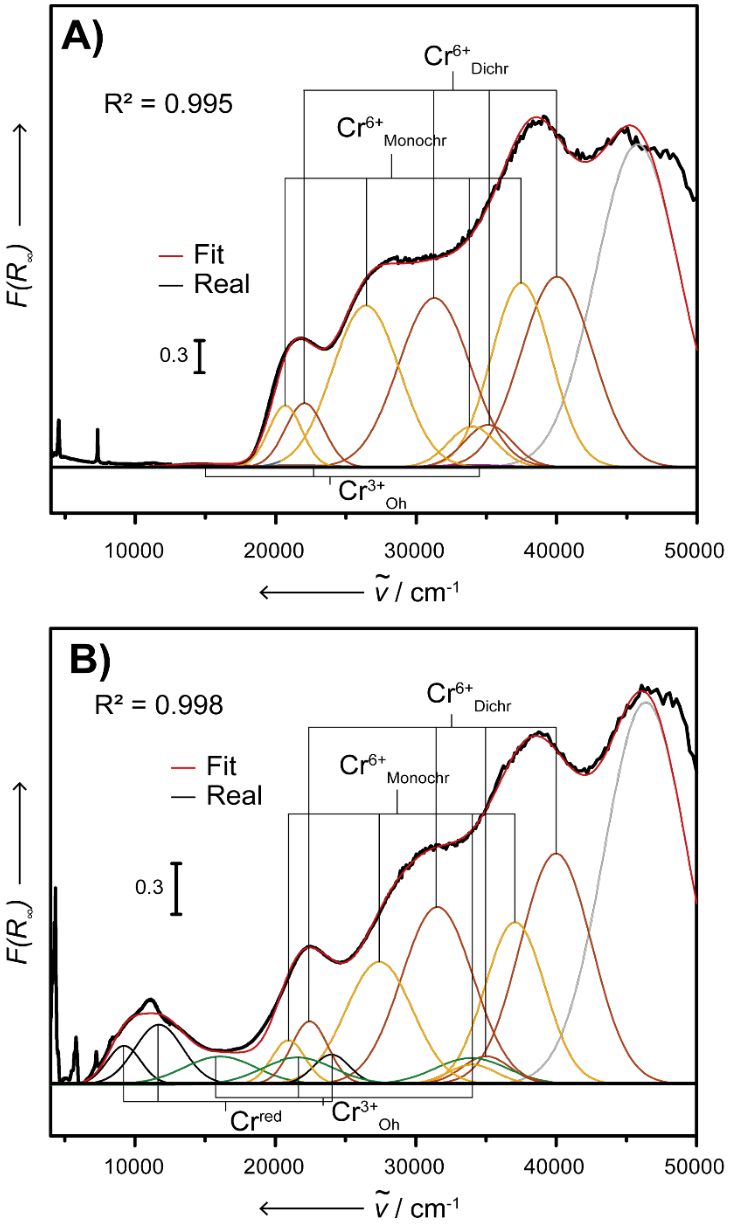

Figure S8. A) Deconvoluted UV-Vis-NIR DRS spectrum of the Cr^{6+}/SiO_2 catalyst material before pre-treatment with 5.0 molecular equivalents of TEB. **B)** Deconvoluted UV-Vis-NIR DRS spectrum after pre-treatment with TEB in a N_2 stream of 10 mL/min, aiming for a B:Cr mole ratio of 5.0. The machinal artefact at 11000 cm^{-1} is not taken into account for deconvolution.

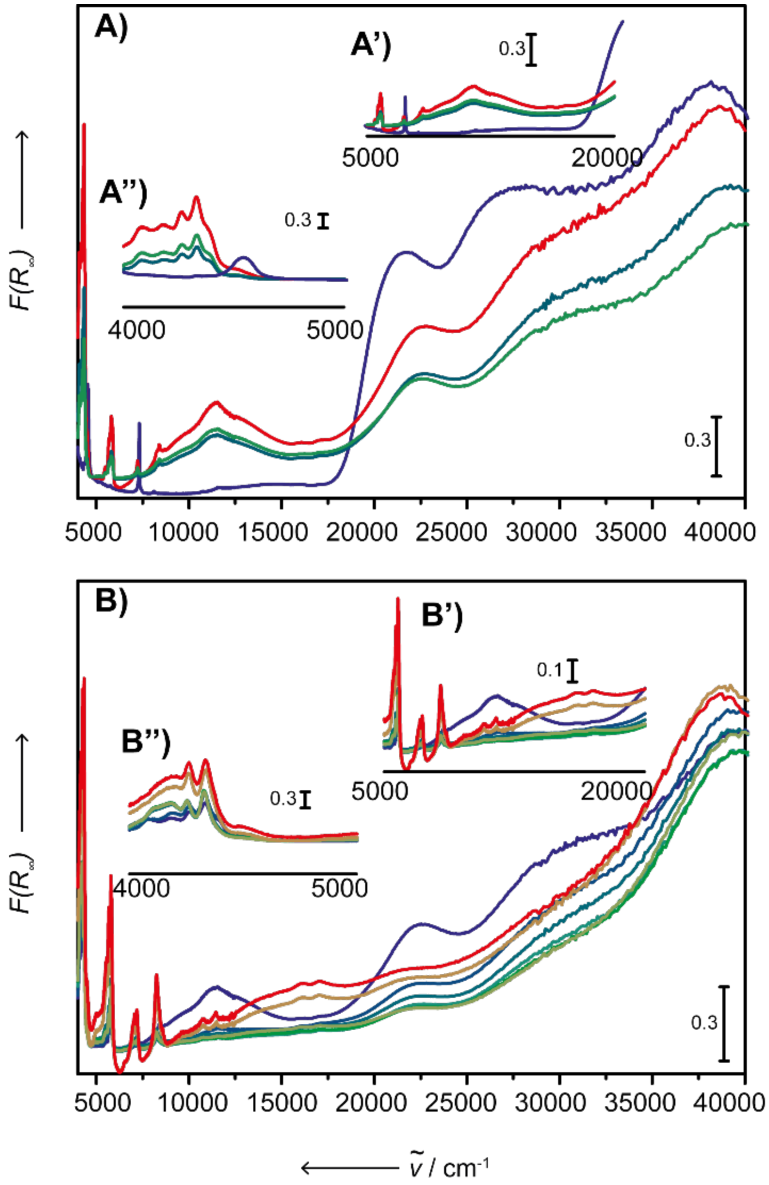

Figure S9. UV-Vis-NIR DRS spectral developments for the experiment with a B:Cr mole ratio of 10.0, from **blue** to **red**, divided in reduction and polymerization. The spectra, from blue to red, are recorded with 5 min intervals. **A)** The pre-treatment of the catalyst with TEB under an N_2 stream of 10 mL/min at room temperature and ambient pressure, aiming for a B:Cr mole ratio of 10. **B)** Spectral developments after the N_2 stream was switched to a C_2H_4 stream of 10 mL/min, where the spectra were recorded at 20 ˚C intervals. The spectra, from blue to red, are recorded with 5 min intervals.

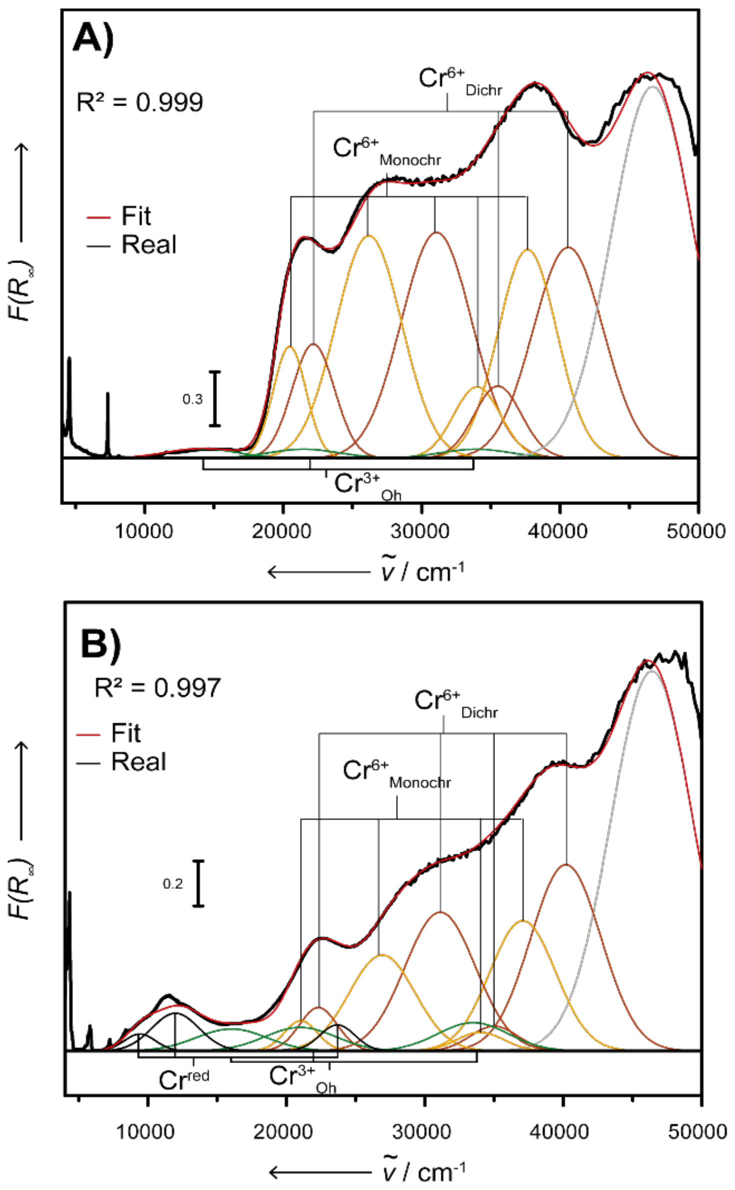

Figure S10. A) Deconvoluted UV-Vis-NIR DRS spectrum of the Cr^{6+}/SiO_2 catalyst material before pre-treatment with 10 molecular equivalents of TEB. **B)** Deconvoluted UV-Vis-NIR DRS spectrum after pre-treatment with TEB in a N_2 stream of 10 mL/min, aiming for a B:Cr mole ratio of 10. The machinal artefact at 11000 cm^{-1} is not taken into account for deconvolution.

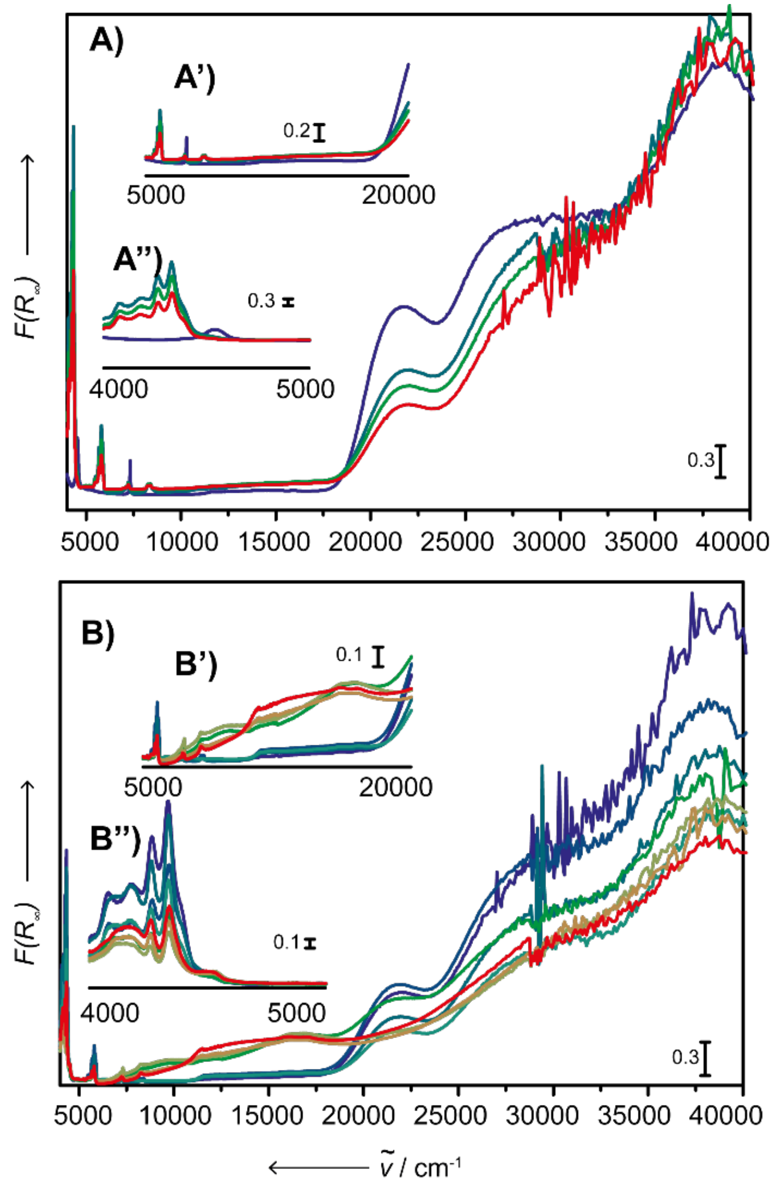

Figure S11. UV-Vis-NIR DRS spectral developments for the experiment with an Al:Cr mole ratio of 1.50, from **blue** to **red**, divided in reduction and polymerization. The spectra, from blue to red, are recorded with 5 min intervals. **A)** the Pre-treatment of the catalyst with TEAl under an N_2 stream of 10 mL/min at room temperature and ambient pressure, aiming for an Al:Cr mole ratio of 1.50. **B)** Spectral developments after the N_2 stream was switched to a C_2H_4 stream of 10 mL/min, where the spectra were recorded at 20 ˚C intervals. The spectra, from blue to red, are recorded with 5 min intervals.

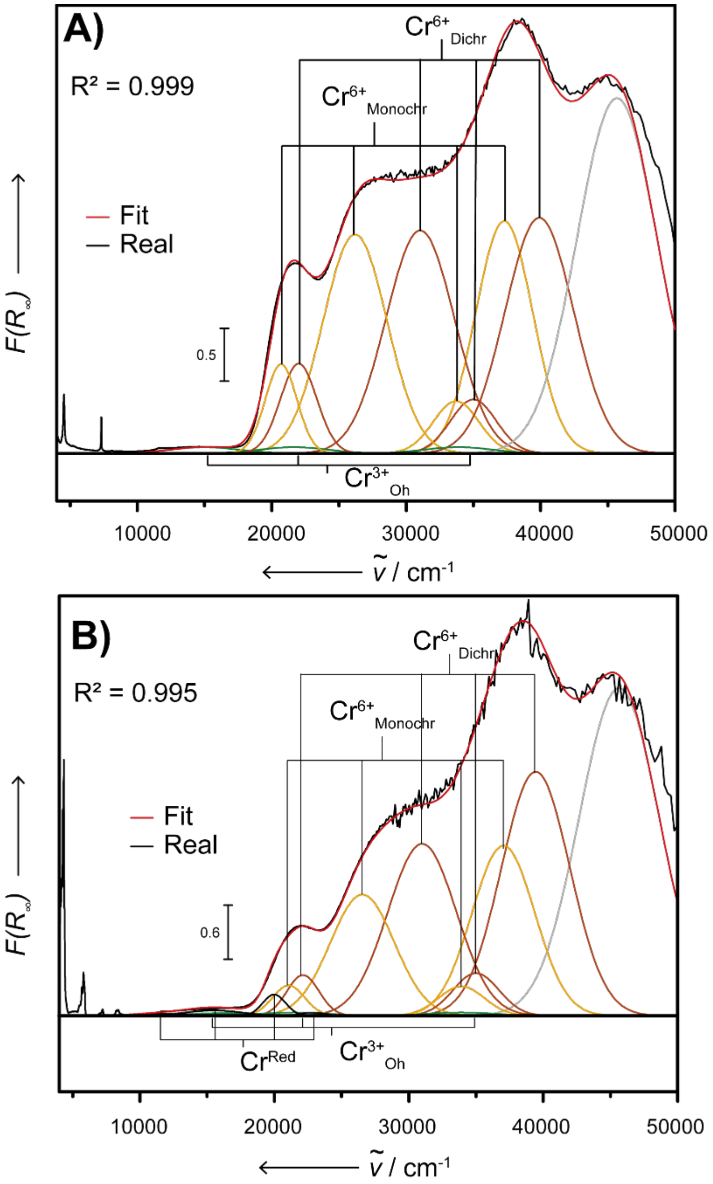

Figure S12. A) Deconvoluted UV-Vis-NIR DRS spectrum of the Cr^{6+}/SiO_2 catalyst material before pre-treatment with 1.50 molecular equivalents of TEAl. **B)** Deconvoluted UV-Vis-NIR DRS spectrum after pre-treatment with TEAl in a N_2 stream of 10 mL/min, aiming for an Al:Cr mole ratio of 1.50. The machinal artefact at 11000 cm^{-1} is not taken into account for deconvolution.

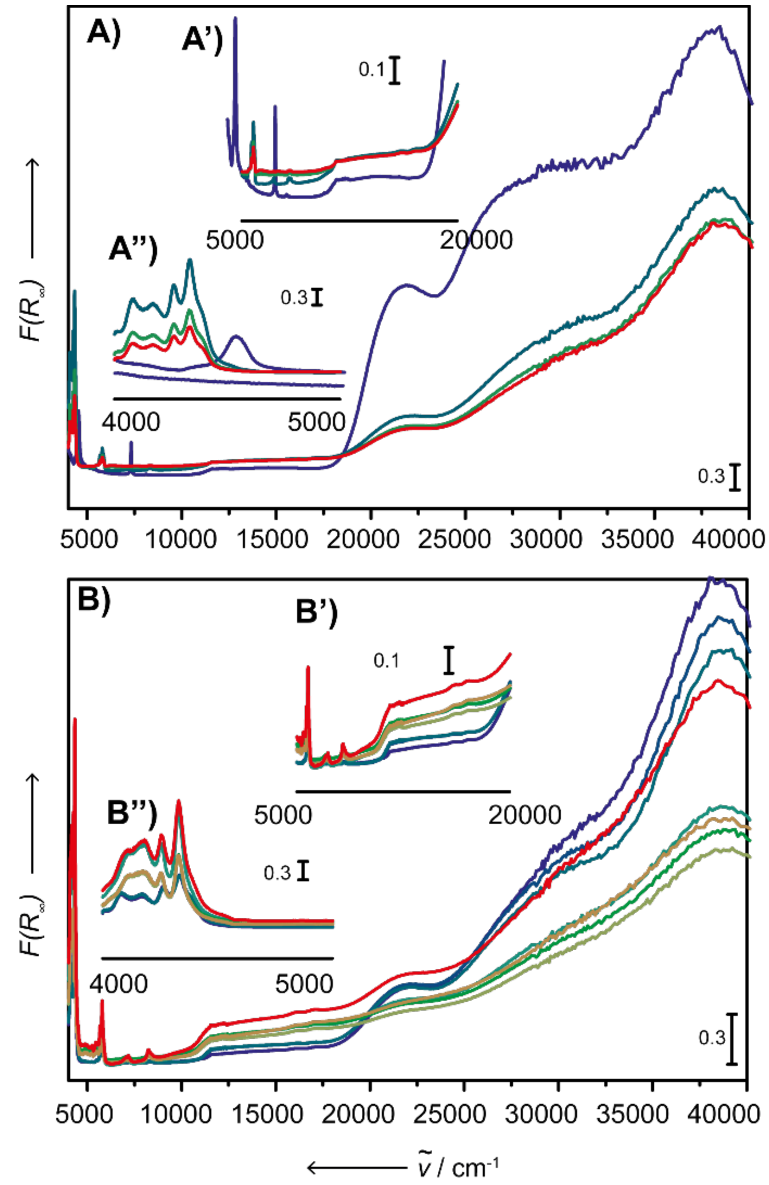

Figure S13. UV-Vis-NIR DRS spectral developments for the experiment with an Al:Cr mole ratio of 5.0, from **blue** to **red**, divided in reduction and polymerization. The spectra, from blue to red, are recorded with 5 min intervals. **A)** The pre-treatment of the catalyst with TEAl under an N_2 stream of 10 mL/min at room temperature and ambient pressure, aiming for an Al:Cr mole ratio of 5.0. **B)** Spectral developments after the N_2 stream was switched to a C_2H_4 stream of 10 mL/min, where the spectra were recorded at 20 ˚C intervals. The spectra, from blue to red, are recorded with 5 min intervals.

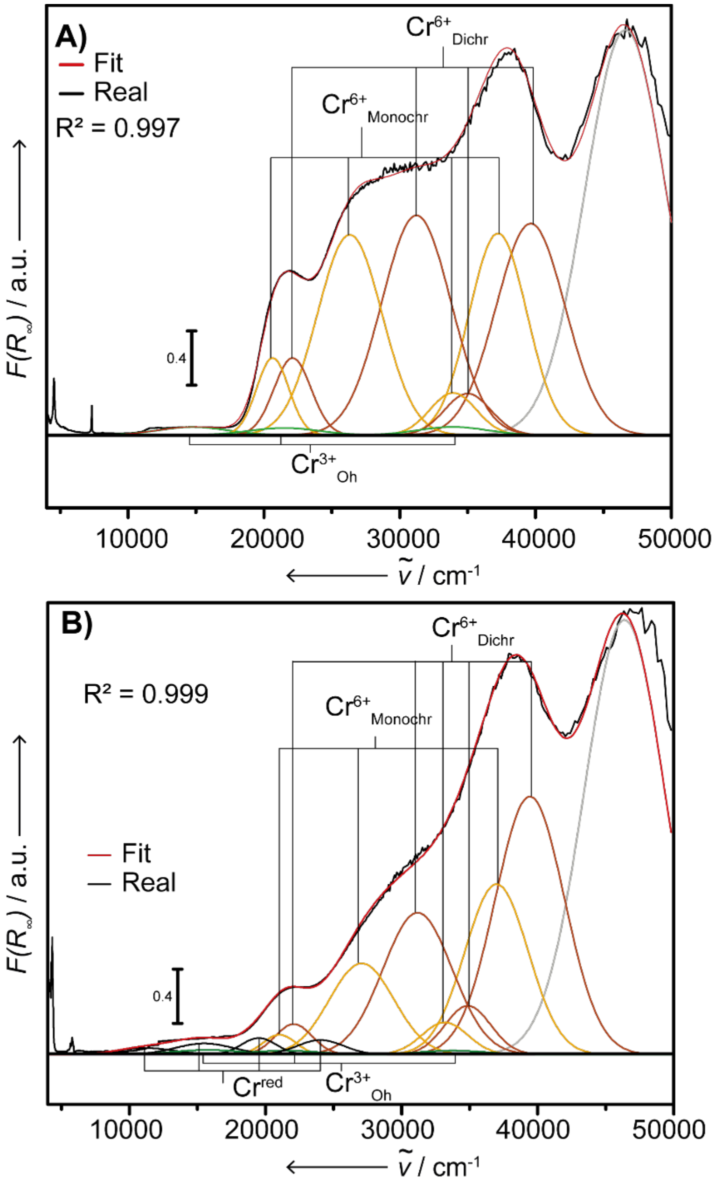

Figure S14. A) Deconvoluted UV-Vis-NIR DRS spectrum of the Cr^{6+}/SiO_2 catalyst material before pre-treatment with 5.0 molecular equivalents of TEAl. **B)** Deconvoluted UV-Vis-NIR DRS spectrum after pre-treatment with TEAl in a N_2 stream of 10 mL/min, aiming for an Al:Cr mole ratio of 5.0. The machinal artefact at 11000 cm^{-1} is not taken into account for deconvolution.

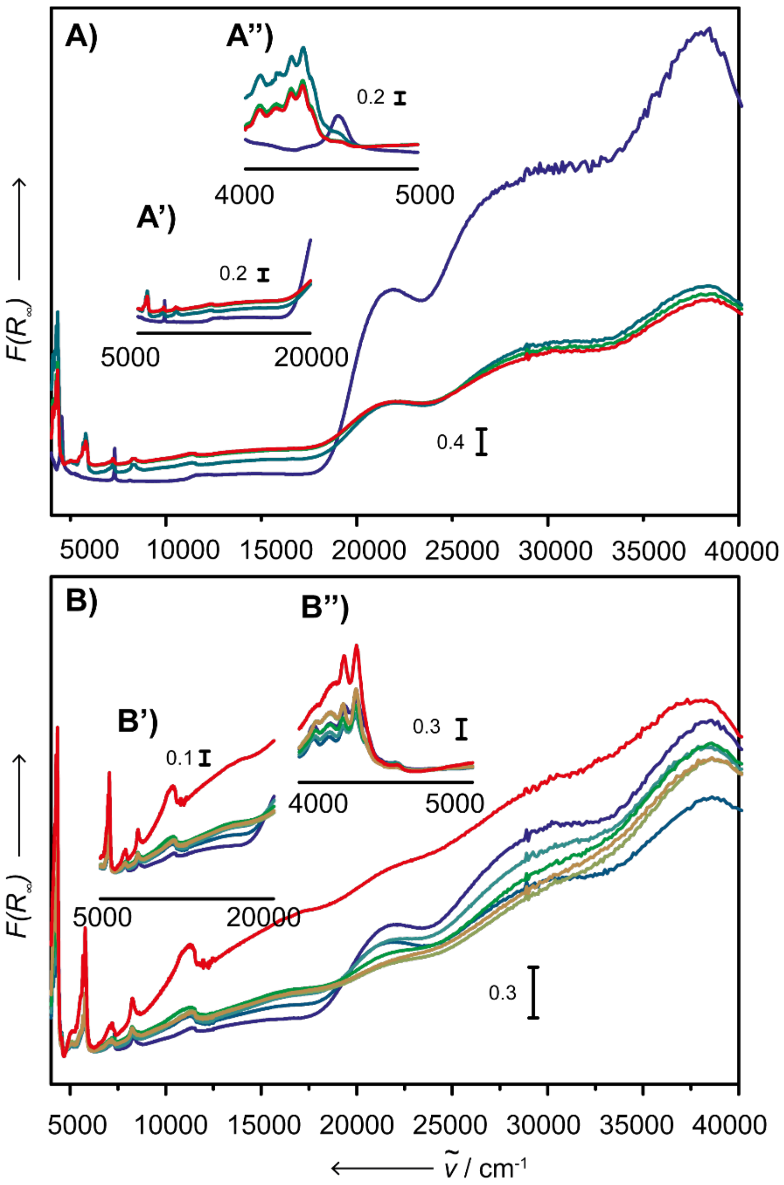

Figure S15. UV-Vis-NIR DRS spectral developments for the experiment with an Al:Cr mole ratio of 10.0, from **blue** to **red**, divided in reduction and polymerization. The spectra, from blue to red, are recorded with 5 min intervals. **A)** The pre-treatment of the catalyst with TEAl under an N_2 stream of 10 mL/min at room temperature and ambient pressure, aiming for an Al:Cr mole ratio of 10. **B)** Spectral developments after the N_2 stream was switched to a C_2H_4 stream of 10 mL/min, where the spectra were recorded at 20 ˚C intervals. The spectra, from blue to red, are recorded with 5 min intervals.

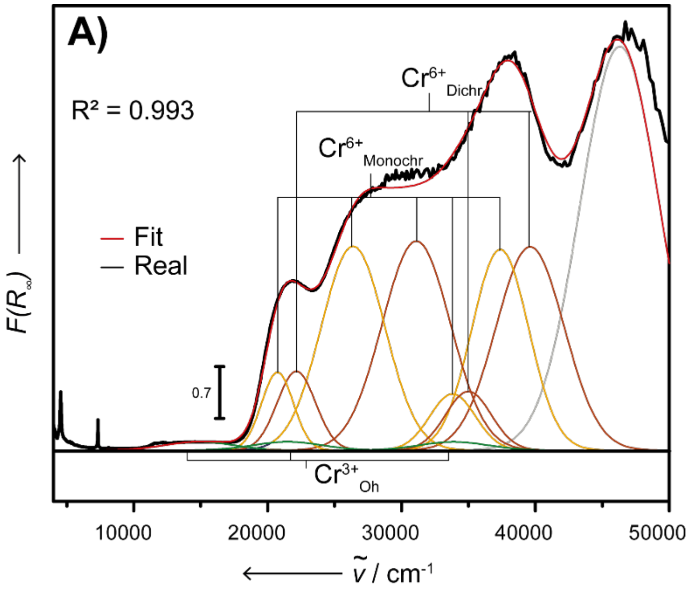

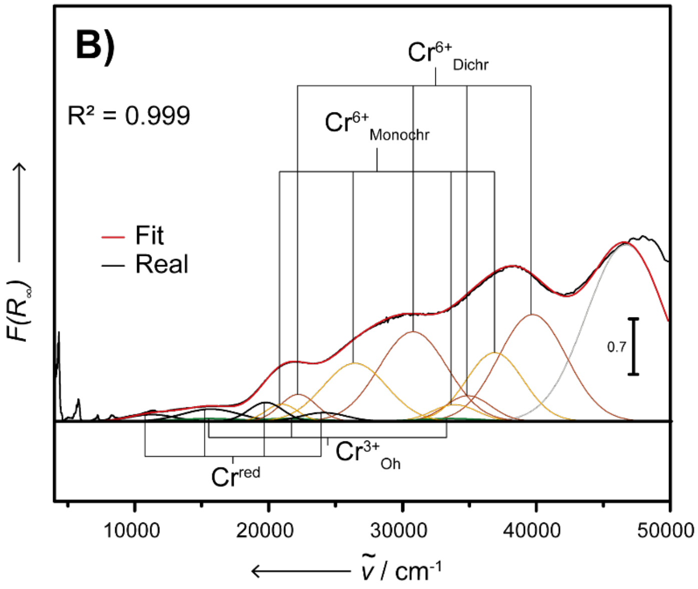

Figure S16. A) Deconvoluted UV-Vis-NIR DRS spectrum of the Cr^{6+}/SiO_2 catalyst material before pre-treatment with 10 molecular equivalents of TEAl. **B)** Deconvoluted UV-Vis-NIR DRS spectrum after pre-treatment with TEAl in a N_2 stream of 10 mL/min, aiming for a Al:Cr mole ratio of 10. The machinal artefact at 11000 cm^{-1} is not taken into account for deconvolution.

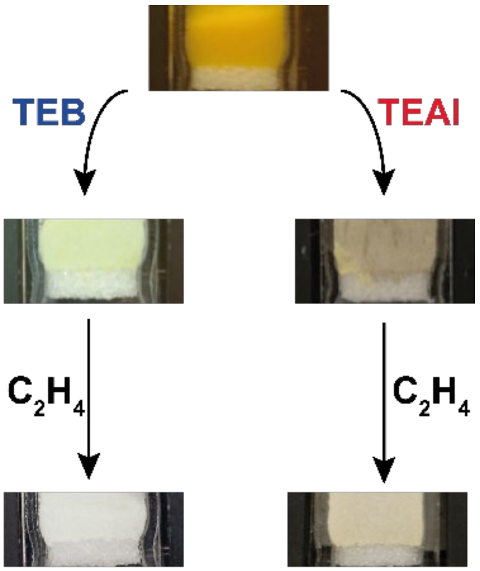

Figure S17. Photographs of the Cr/SiO_2 catalyst in the quartz reaction cell before reduction, after reduction with TEB or TEAl and after ethylene polymerization.

Additional References

[1] P.J. DesLauriers, D.C. Rohlfing, *Macromol. Symp.* **2009**, *282*, 136-149
